# Chemical stimuli override a temperature-dependent morphological program by reprogramming the transcriptome of a fungal pathogen

**DOI:** 10.1128/mbio.02234-25

**Published:** 2025-09-10

**Authors:** Dror Assa, Mark Voorhies, Anita Sil

**Affiliations:** 1 Department of Microbiology and Immunology, University of California San Francisco 214560, San Francisco, California, USA; 2 Chan Zuckerberg Biohub–San Francisco 578083, San Francisco, California, USA; Instituto Carlos Chagas, Curitiba, Brazil

**Keywords:** fungal pathogenesis, hyphal development, transcription factors

## Abstract

The human fungal pathogen *Histoplasma* changes its morphology in response to temperature. At 37°C, it grows as a budding yeast, whereas at room temperature (RT), it transitions to hyphal growth. Prior work has demonstrated that 15–20% of transcripts are temperature-regulated, and that transcription factors (TFs) Ryp1-4 are necessary to establish yeast growth. However, little is known about transcriptional regulators of the hyphal program. To identify TFs that regulate filamentation, we utilize chemical inducers of hyphal growth. We show that the addition of cAMP analogs or an inhibitor of cAMP breakdown overrides yeast morphology, yielding inappropriate hyphal growth at 37°C. Additionally, butyrate supplementation triggers hyphal growth at 37°C. Transcriptional profiling of cultures filamenting in response to cAMP or butyrate reveals that a limited set of genes responds to cAMP, while butyrate dysregulates a larger set. Comparison of these profiles to previous temperature- or morphology-regulated gene sets identifies a small set of morphology-specific transcripts, including nine TFs. We characterized three TFs, *STU1*, *FBC1*, and *PAC2*, whose orthologs regulate development in other fungi. We found that while only *FBC1* is necessary for RT-induced filamentation, each of these TFs is required for other aspects of RT development. Additionally, *FBC1* and *PAC2*, but not *STU1*, are necessary for filamentation in response to cAMP at 37°C. Ectopic expression of each of these TFs is sufficient to induce filamentation at 37°C and *PAC2* induction of filamentation at 37°C is dependent on *STU1*. Taken together, this work identifies filamentation-promoting TFs that form regulatory circuits to promote the hyphal program.

**IMPORTANCE:**

Fungal illnesses pose a significant disease burden. However, the regulatory circuits that govern fungal development remain largely unknown. This study utilizes chemicals that can override the normal growth morphology of the human pathogen *Histoplasma*. Using transcriptomic approaches, we identify novel regulators of the hyphal morphology and refine our understanding of the transcriptional circuits governing morphology in *Histoplasma*.

## INTRODUCTION

To survive in diverse environments, microbes employ a variety of mechanisms to sense and respond to distinct signals, allowing them to adapt to different conditions. Thermally dimorphic fungi have developed the ability to thrive in diverse conditions, growing as hyphae at ambient temperatures and at room temperature (RT) in the laboratory, but switching to a unicellular growth form at mammalian body temperature (37°C in the laboratory). *Histoplasma ohiense* (previously described as *H. capsulatum*) is one such thermally dimorphic fungus that grows as conidiating hyphae in the soil and, after inhalation, changes its shape and gene expression program to generate virulent yeast cells in response to mammalian body temperature. This transition to yeast cells can result in an acute pulmonary infection known as histoplasmosis (1, 2). Overall, morphological differentiation is key to *Histoplasma* biology, requiring the organism to integrate signals on a molecular level and convert those signals into transcriptional, translational, and biochemical changes that orchestrate the transition from one growth form to another.

The molecular basis of the ability of *Histoplasma* to transition between morphologies has been the focus of many studies in the past decades. Previous work had shown that 15–20% of transcripts in *Histoplasma* are regulated by temperature (3–5). Specific functions, such as virulence, iron acquisition, and cell wall modification, are correlated with 37°C and yeast-phase growth, whereas the hyphal growth form that dominates at RT promotes the expression of enzymes such as tyrosinases, cytochrome p450s, oxidoreductases, and peroxidases. Additionally, the abundance of *Histoplasma* transcripts encoding 18 putative transcription factors (TFs) is increased in hyphae compared to yeast (3).

Forward genetic screens have been instrumental in finding regulators of morphology, identifying Drk1, a hybrid histidine kinase orthologous to *Candida albicans* Nik1 (6), which is necessary for yeast morphology (7), and three TFs that are also required for yeast phase (RYP) growth: Ryp1, a WOPR family TF orthologous to *C. albicans* Wor1, and Ryp2/3, both velvet family TFs orthologous to *Aspergillus* VosA and VelB, respectively (8, 9). Follow-up studies also identified an additional TF RYP growth, Ryp4, a Zn(II)2Cys6 zinc binuclear cluster domain protein (4). Furthermore, Gilmore et al. showed that inappropriate expression of the TF Wet1 is sufficient to trigger filamentous growth at 37°C in *Histoplasma* (3). Wet1 is orthologous to wetA in *Aspergillus*, which regulates the late stages of asexual spore production (10). More recently, Rodriguez et al. reported that deregulated expression of the APSES-family TF Stu1, which is orthologous to StuA in *Aspergillus* and Enhanced Filamentous Growth 1 (Efg1) in *C. albicans*, is also sufficient to trigger inappropriate filamentous growth at 37°C in *Histoplasma* (11, 12). Additionally, Longo et al. showed that using RNA interference to target Stu1 resulted in decreased formation of aerial hyphae on plates (13). StuA coordinates conidiophore formation in the *Aspergilli* (14, 15), and Efg1 is necessary for filamentous growth in *C. albicans* (reviewed in reference 16).

Here, we examine the relationship between transcript abundance, *Histoplasma* hyphae formation, and the cAMP/protein kinase A (PKA) pathway, which is a central and conserved signaling pathway in all eukaryotes. In fungi, the cAMP-PKA pathway regulates carbon utilization, mating, morphology, stress response, and virulence. The cAMP-PKA pathway has been the subject of extensive research in *S. cerevisiae* (reviewed in references 17–19). In this organism, fermentable carbon sources such as glucose are sensed by several pathways that lead to the activation of adenylate cyclase, which synthesizes cAMP from ATP. cAMP then binds the regulatory subunits of PKA, releasing the catalytically active subunits to phosphorylate target proteins. One of the main roles of the cAMP-PKA pathway in *S. cerevisiae* is to allow rapid response to stress. In non-stress conditions, the TFs Msn2/4p are phosphorylated by PKA, preventing them from entering the cell nucleus (20). Various stress signals inactivate PKA rapidly and cause the induction of the environmental stress response (21–25). Other outcomes of PKA inactivation include induction of gluconeogenesis, respiration, and alternative energy source utilization pathways and repression of cell division and ribosomal biogenesis pathways (26, 27). The cAMP-PKA pathway also regulates pseudohyphal growth by activating the TF Flo8p (28, 29). In *Candida* species, the cAMP-PKA pathway integrates signals such as environmental pH, N-acetylglucosamine (GlcNAc), and the presence of serum in growth media and, depending on specific contexts, positively regulates white-opaque switching or the transition to hyphal growth, mediated by the activation of Efg1 (30–37). However, recent studies have indicated that the cAMP/PKA pathway is not strictly necessary for the induction of filamentation (32, 33, 38). The activity of the cAMP-PKA pathway is also necessary for the expression of virulence genes in *C. albicans* (35, 39, 40). In *Aspergillus* spp., sensing of signals such as glucose by hetero-trimeric G-protein leads to activation of the cAMP-PKA pathway, inducing spore germination, inhibiting sporulation, and coordinating the synthesis of secondary metabolites (SMs) (41–46). Finally, intracellular and extracellular cAMP levels rise when *Histoplasma* G217B yeast (growing at 37°C) is shifted to RT, concurrent with the formation of hyphae (47). These data suggest a correlation between cAMP accumulation and filamentation. Moreover, the addition of the stable cAMP analog dibutyryl-cAMP (dbcAMP) to *Histoplasma* cultures growing at 37°C promotes filamentous growth (47–49). Similarly, in the closely related dimorphic fungi *Paracoccidioides brasiliensis* and *Blastomyces dermatitidis*, exogenous cAMP delays the transition from hyphae to yeast (50, 51). However, the underlying molecular mechanisms of filamentation induced by exogenous activation of the cAMP pathway in thermally dimorphic fungi have not been examined.

In this study, we used transcriptional profiling of *Histoplasma* to gain insight into the molecular events that occur upon chemical induction of filamentation. By comparing these expression profiling experiments to RT-induced expression, we identified groups of transcripts whose accumulation is associated with yeast or hyphal morphologies. Finally, we focused on three TFs that have been shown to regulate morphology and development in other organisms and showed that they are capable of driving a hyphal program in *Histoplasma*.

## RESULTS

### cAMP analogs drive inappropriate filamentation at 37°C

**Fig 1:**
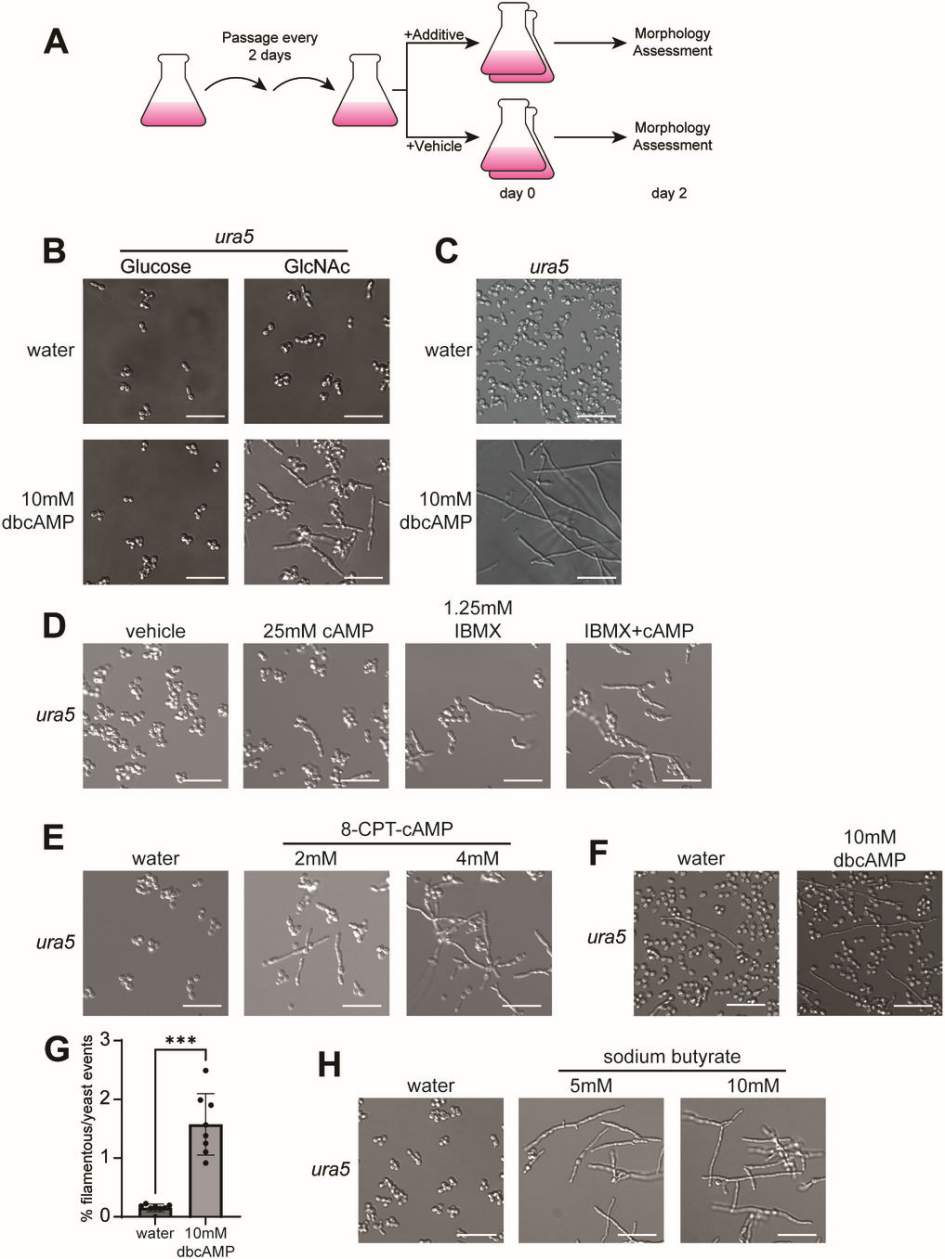
Exogenous cAMP promotes filamentous growth at 37°C. (**A**) Schematic outline of experiments testing the response of *Histoplasma* to chemical additives. (**B**) Cell morphology of G217B and G217B*ura5 Histoplasma* after 2 days of growth at 37°C in liquid HMM or HMM/GlcNAc with or without 10 mM dbcAMP. (**C**) Cell morphology of G217B*ura5 Histoplasma* after 8 days of growth on HMM/GlcNAc-agarose plates, with or without 10 mM dbcAMP, at 37°C. Cells were scraped off the plates and suspended on a slide for imaging. (**D**) Cell morphology of G217B*ura5 Histoplasma* after 2 days of growth at 37°C in liquid HMM/GlcNAc with 1.25 mM IBMX, 25 mM cAMP, IBMX + cAMP, or water as vehicle control. (**E**) Cell morphology of G217B*ura5 Histoplasma* after 2 days of growth at 37°C in liquid HMM/GlcNAc with 4 mM or 2 mM 8-CPT-cAMP, or water as vehicle control. (**F**) Cell morphology of G217B*ura5 Histoplasma* after 7 days of growth at RT in liquid HMM containing glucose with or without 10 mM dbcAMP. (**G**) Quantification of filamentation after 7 days at RT in liquid HMM containing glucose with or without 10 mM dbcAMP; *N* > 243 events. (**H**) Cell morphology of G217B*ura5 Histoplasma* after 2 days of growth at 37°C in liquid HMM/GlcNAc with 5 or 10 mM sodium butyrate, or water as vehicle. Scale bar denotes 10 µm; ***P < 0.001, Wilcoxon rank-sum test.

To characterize the effect of dbcAMP on *Histoplasma* growth, we added dbcAMP to early-log phase G217B and G217B*ura5* (WU15) cultures growing at 37°C in HMM or HMM/GlcNAc (see Materials and Methods) (Fig. 1A). Our previous work showed that GlcNAc stimulates hyphal growth upon shift from 37°C to RT (52). While no morphological difference was observed after 1 day, within 2 days, a noticeable increase in filamentous growth occurred in cultures growing in HMM/GlcNAc at 37°C and treated with dbcAMP compared to the vehicle (water). In contrast, 10 mM dbcAMP did not affect the morphology of yeast cultures in HMM containing only glucose at 37°C (Fig. 1B; Fig. S1A). Hyphal growth was even more evident by the third day after dbcAMP addition (Fig. S1A and B). The extent to which dbcAMP promotes filamentation was concentration-dependent: a visible increase in filamentous growth could be observed in cultures with as little as 1.25 mM dbcAMP (Fig. S1C). Addition of dbcAMP to solid media (HMM/GlcNAc agarose) also strongly favored filamentous growth at 37°C (Fig. 1C).

We next wanted to test whether other means of increasing cAMP levels had a similar effect on hyphae formation. Normally, cAMP is broken down within the cell by the action of phosphodiesterases. We attempted to raise cAMP levels by adding cAMP sodium salt or the phosphodiesterase inhibitor IBMX. Whereas 25 mM cAMP had a negligible effect on morphology on its own, the addition of 1.25 mM IBMX was sufficient to trigger filamentation within 2 days (Fig. 1D). Adding both cAMP and IBMX had a stronger filamentation effect, suggesting that minimizing the breakdown of exogenous cAMP by inhibiting phosphodiesterase activity may cause a net increase in cAMP levels that promotes filamentation. Furthermore, adding 8-(4-chlorophenylthio)adenosine 3′,5′-cyclic monophosphate (8-CPT-cAMP), an alternative cAMP analog, also promoted robust filamentation (Fig. 1E). Taken together, these data confirm that exogenously added cAMP overrides yeast phase growth and promotes filamentation at 37°C.

The transition from yeast to hyphae in response to temperature shift is less robust in glucose relative to GlcNAc (52). To determine if the addition of exogenous cAMP promotes filamentation in response to temperature shift in glucose, where the transition is asynchronous and inefficient, we added 10 mM dbcAMP to cultures shifted from 37°C to RT in glucose-containing media. We observed a subtle (Fig. 1F) but statistically significant (Fig. 1G) increase in elongated cells and filamentous structures in liquid culture by day 7 after shift in the presence of dbcAMP.

Since dbcAMP hydrolysis can release butyrate into the growth media, we also investigated the effect of adding sodium butyrate on its own to *Histoplasma* growth media. In the presence of GlcNAc, butyrate strongly induced filamentation within 2 days; however, the effect of butyrate was much more potent than dbcAMP. For example, 10 mM dbcAMP stimulated robust filamentation by day 2 (Fig. 1B), but yeast cells were still present. In contrast, 5 mM butyrate stimulated complete hyphal transformation with no yeast cells remaining in the cultures (Fig. 1H, S1D). The increased potency of butyrate compared to dbcAMP suggested that butyrate stimulates filamentation by a separate mechanism from dbcAMP. Additionally, the robust stimulation of filamentation by butyrate suggested that it could be another useful tool to identify pathways that are linked to driving the hyphal program.

### cAMP analogs and butyrate stimulate robust transcriptional changes

To further understand the molecules and pathways that mediate filamentation after addition of cAMP analogs or butyrate, we performed transcriptional profiling of G217B*ura5 Histoplasma* cultures grown in HMM/GlcNAc media at 37°C with addition of 10 mM dbcAMP, 4 mM 8-CPT-cAMP, 5 mM butyrate, or water as vehicle control. Each of these additions stimulated filamentation compared to the water control (Fig. S2A). We found that 721 or 1,083 transcripts were significantly increased in abundance in response to dbcAMP or 8-CPT-cAMP, respectively (>1.5-fold change, FDR < 5%) and 639 or 1,000 transcripts were decreased in abundance in response to dbcAMP or 8-CPT-cAMP, respectively. In comparison, the response to butyrate involved more genes: 2,091 transcripts increased in abundance in response to butyrate, and 2,383 transcripts decreased in abundance.

**Fig 2:**
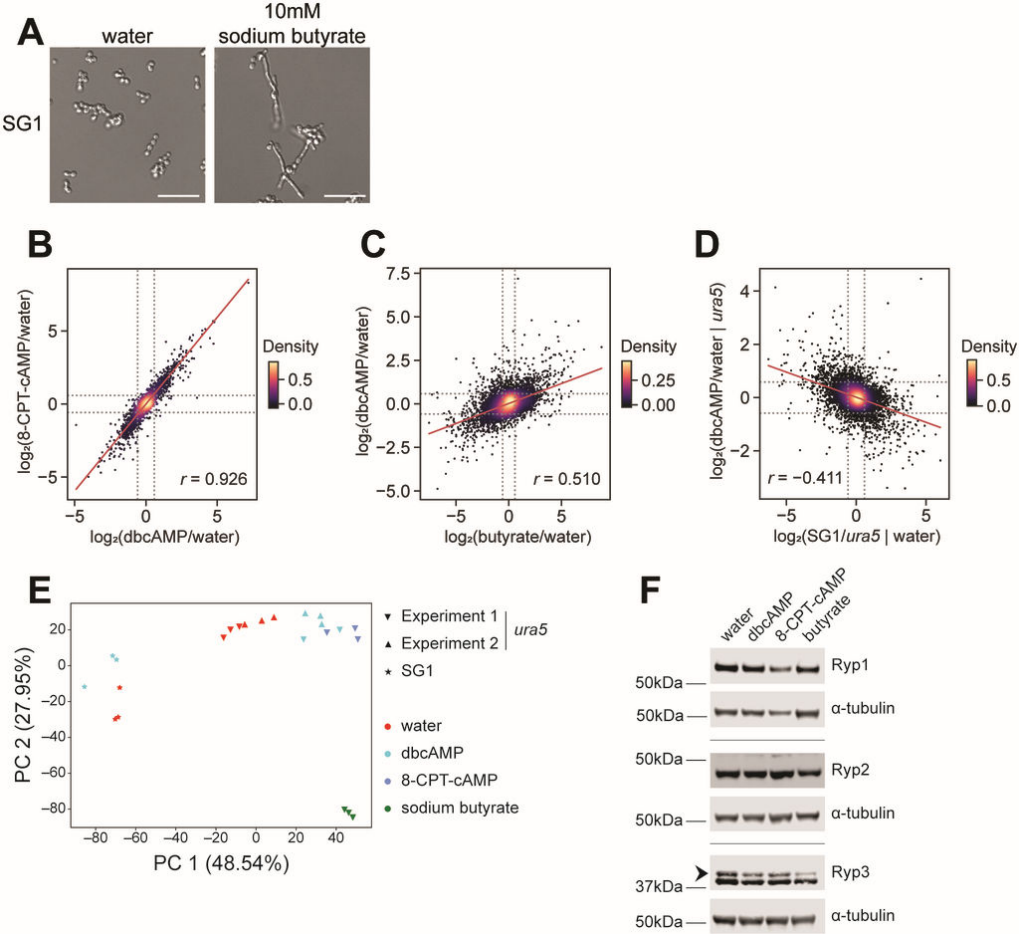
RNAseq revealed the transcriptional response to cAMP analogs and butyrate. (**A**) Cell morphology of SG1 *Histoplasma* after 2 days of growth at 37°C in liquid HMM/GlcNAc with 10 mM sodium butyrate or water as vehicle control. (**B, C**) Scatter plots of LIMMA-fit treatment/vehicle contrasts log_2_ ratios; dashed line indicates fold change of log_2_1.5. (**D**) Scatter plot of LIMMA-fit contrasts of log_2_ ratios for genotype (with vehicle) versus treatment (in G217B*ura5*); dashed line indicates fold change of log_2_1.5. (**E**) Principal component analysis (PCA) of all genes expressed in G217B*ura5* and SG1 *Histoplasma* treated with dbcAMP, 8-CPT-cAMP, sodium butyrate, or water. (**F**) Western blots performed on protein extracted from G217B*ura5 Histoplasma* after 2 days of growth at 37°C in liquid HMM/GlcNAc with 10 mM dbcAMP, 4 mM 8-CPT-cAMP, 5 mM butyrate, or water as vehicle control; arrow denotes Ryp3 band. Scale bar denotes 10 µm; *r*, Pearson’s correlation coefficient.

A previous forward genetic screen for yeast-locked mutants identified SG1, a WU15-derived strain that is refractory to filamentous growth in response to acute RT shift (11, 12). In contrast to its parental G217B*ura5* strain, SG1 did not filament in response to 10 mM dbcAMP at 37°C (Fig. S2B). However, SG1 did filament in response to 10 mM butyrate (Fig. 2A), albeit to a lesser extent than the parental strain (Fig. S2A), highlighting the differential effects of dbcAMP and butyrate addition. Given the distinct morphological response of SG1 to dbcAMP, we performed a second transcriptional profiling experiment comparing the responses of G217B*ura5* and SG1 yeast to dbcAMP treatment. The normalized transcript counts in corresponding conditions in the two experiments, that is, G217B*ura5* treated with vehicle (water; Fig. S2C) or 10 mM dbcAMP (Fig. S2D) exhibited very high correlation (*r* = 0.99), indicating that the two experiments are comparable.

We next compared the effect of dbcAMP on the transcriptome of G217Bura5 to that of 8-CPT-cAMP and butyrate. The effects of cAMP analogs are highly correlated (Fig. 2B, Pearson’s *r* = 0.9). In contrast, the transcriptional response to butyrate is overlapping but distinct (Fig. 2C, *r* = 0.5). The transcriptional changes due to the SG1 genotype are weakly anticorrelated to the effect of dbcAMP on G217B*ura5* (Fig. 2D, *r* = −0.4), indicating that SG1 responds differently than G217B*ura5* to cAMP analogs transcriptionally as well as morphologically. Simultaneously considering the correlations among all of the transcriptional profiles with principal component analysis (PCA) reveals a first component, representing half of the total variance, that captures the correlated response to dbcAMP and butyrate, anticorrelated with the effects of the SG1 genotype, and a second component, representing half of the remaining variance, capturing an orthogonal response unique to butyrate (Fig. 2E).

**Fig 3:**
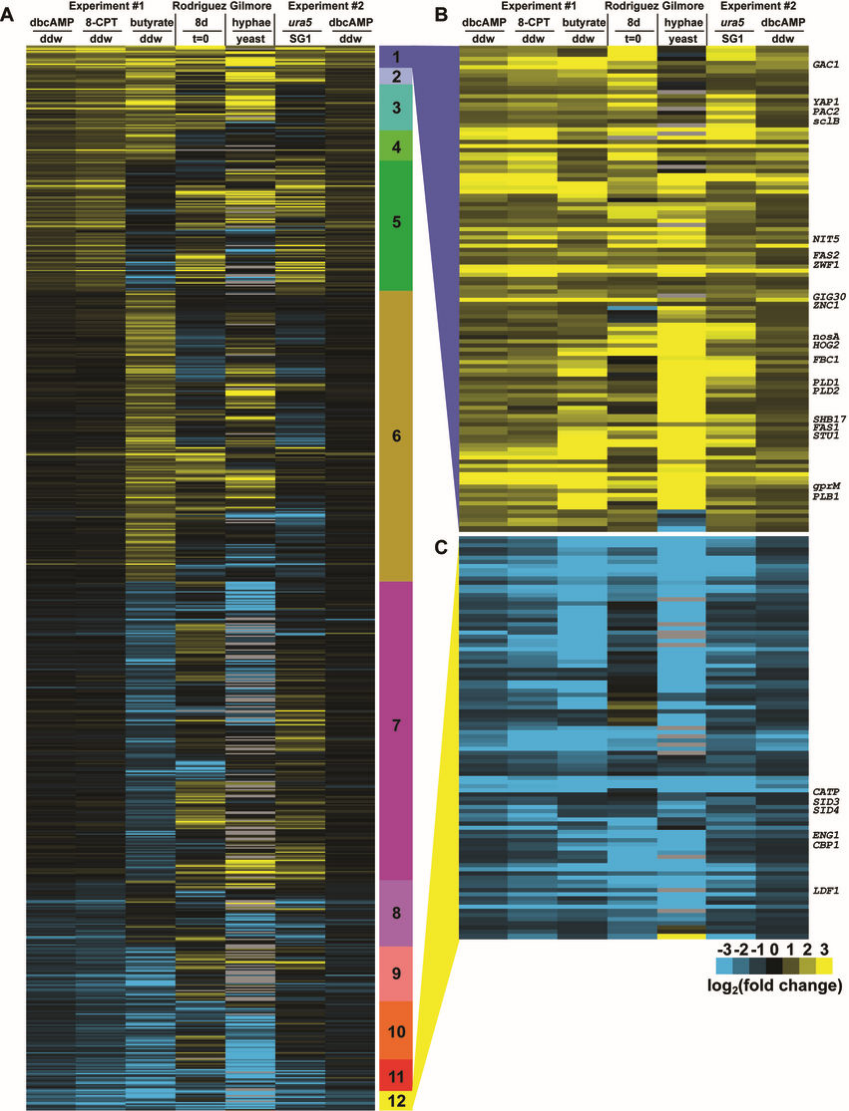
Genetic and environmental perturbations define high-confidence morphology regulons. (**A**) Heatmap showing 5,494 genes differentially expressed in response to butyrate, dbcAMP, or 8-CPT-cAMP (Table S3). Genes are grouped by expression pattern according to the scheme in Fig. S3 as indicated by the colored bar to the right of the heatmap. Heatmap colors indicate log_2_ ratios of cpm values. (**B**) Expanded view of the high-confidence yeast regulon (Class 1, 112 genes; Table S5). (**C**) Expanded view of the high-confidence hyphal regulon (Class 12, 93 genes; Table S6).

To stringently define high-confidence regulons specific to yeast or hyphal morphology, we progressively gated the transcriptome on expression values that change when the cells are given a filamentation signal in a manner that is dependent on genotype (wild-type or SG1) (Fig. S3). 1,270 transcripts were upregulated (i.e., showed increased abundance) in response to either cAMP analog. Of these, 594 were also upregulated in response to butyrate. Of these, 400 were also induced at RT in at least one of the two previously published RT experiments. Of these, 196 were downregulated (i.e., showed decreased abundance) in the non-filamenting SG1 mutant. Finally, 112 of these transcripts were also down in response to dbcAMP in the SG1 experiment, and we designated this set as the stringently defined hyphal-associated set (Class 1 in Fig. 3A and B; Fig. S3). Likewise, 1,186 transcripts were downregulated in response to either cAMP analog. Of these, 842 were also downregulated in response to butyrate. Of these, 481 were also repressed at RT in at least one of the previously published RT experiments. Of these, 237 were upregulated in the non-filamenting SG1 mutant. Finally, a high-confidence yeast-associated subset of 93 of these genes was also up in response to dbcAMP in the SG1 experiment. We designated this set as the stringently defined yeast-associated set (Class 12 in Fig. 3A and C; Fig. S3).

The stringently defined yeast-associated set includes the known virulence factors *CBP1* (53–56), *CATP* (57, 58), *ENG1* (59), *LDF1* (54), and the siderophore biosynthetic genes *SID3* and *SID4* (60). The siderophore biosynthetic cluster as a whole is more downregulated in cAMP than in butyrate, with the remaining genes in the cluster passing our significance criteria only for cAMP. More broadly, genes downregulated in response to butyrate, cAMP, and RT include the additional virulence factor *SOD3* (61); regulator of bud site selection *RSR1*; yeast cell wall-associated enzymes *AMY3* (62) and *CTS1*/*CHS1*; and GH17/*CFP4*, encoding a secreted yeast factor of unknown function (63, 64). The ER chaperones *KAR2* (BiP), *LHS1*, and *CNE1* (calnexin) are likewise downregulated, as is *OCH1*, an initiator of N-linked glycosylation. This group is also enriched for genes whose promoters are bound by Ryp1 (*P* = 7.5e−3), Ryp2 (*P* = 1.7e−4), and Ryp3 (*P* = 7.5e−4, Benjamini-Hochberg corrected hypergeometric test), the TFs that drive yeast-phase growth.

In contrast, the stringently defined hyphal-associated set (Class 1 in Fig. 3A and B) includes anabolic enzymes associated with lipid and riboneogenesis, previously identified hyphal-specific factors, and a variety of signaling molecules, of which nine are TFs. Zamith-Miranda et al. have annotated 77 lipid metabolic genes in *Histoplasma capsulatum* G186AR (65), 71 of which have G217B orthologs with detectable transcripts in our data set. These genes are significantly upregulated in butyrate versus water (*P* = 1.3e−5) and to a lesser extent in cAMP versus water (*P* = 4.9e−4, Wilcoxon rank-sum test). In particular, we do not observe any of the lipid metabolic genes downregulated in response to dbcAMP, only one downregulated in response to 8-CPT-cAMP, and only three down in response to butyrate. Lipid metabolic genes upregulated only in butyrate include the ergosterol pathway. Lipid metabolic genes upregulated in Class 1 include the core fatty acid synthase (FAS1 and FAS2) as well as an elongase (ELO2/GIG30). It may be that increased lipid production modulates hyphal membrane fluidity at RT and thus is coupled to the hyphal program even when filamentation is inappropriately induced at 37°C by chemical stimuli.

The reducing potential to drive fatty acid biosynthesis can be derived from isocitrate dehydrogenase or the oxidative arm of the pentose phosphate pathway (66). While isocitrate dehydrogenase (*IDH1*) is not differential in our data set, *ZWF1*, which catalyzes the first committed step of the oxidative pentose phosphate pathway, yielding NADPH, is in class 1. *GND1*, which catalyzes the other NADPH-generating step of this pathway, is upregulated in butyrate but not cAMP. Intriguingly, *SHB17*, which catalyzes the first committed step in the non-oxidative arm of the pentose phosphate pathway, is also in Class 1. Therefore, these conditions are generating both reducing potential to drive fatty acid biosynthesis, via *ZWF1*, as well as riboneogenesis via *SHB17*, possibly due to increased protein synthesis to replace yeast-specific proteins with hyphal-specific proteins during the transition. While we do not detect upregulation of translational machinery in Class 1, gene ontology (GO) enrichment analysis reveals that 200 ribosome, pre-ribosome, or rRNA processing genes, as well as 28 tRNA processing genes are upregulated in butyrate (Class 6). In addition to its roles in lipid biosynthesis and ribogenesis, we note that *Saccharomyces* null mutants of *ZWF1* require an organic sulfur source to grow (67). Therefore, our observed hyphal-specific expression of *ZWF1* is interesting given the known cysteine auxotrophy of *Histoplasma* yeast but not hyphae (68). On a related note, we see upregulation of the sulfur assimilation pathway in response to filamentation: the first and last genes (*SUL1* and *MET17A*) are in class 2, another (*MET10*) is upregulated in butyrate, and the remainder (*MET3*, *MET14*, *MET16*, and *MET5*) are upregulated in butyrate versus water, cAMP versus water, and RT/37°C but not downregulated in SG1/G217B*ura5* (Class 3). Additionally, 15 genes involved in amino acid synthesis pathways are upregulated in response to dbcAMP.

In addition to the annotated lipid genes (65), we note three distinct phospholipases in Class 1. One phospholipase B (*PLB1*) and one phospholipase D (*PLD1*) have plausible roles in signaling. In particular, the *PLD1* homolog *SPO14* has regulatory roles in meiosis and sporulation in Saccharomyces. Edwards et al. previously noted higher expression of *PLB1* and *PLD1* in G186AR versus G217B yeast (5). The third phospholipase, *PLD2*, which has a conserved secretion signal sequence, is from a distinct lineage of phospholipase D toxins; consistent with toxin function, the *Coccidioides* ortholog of *PLD2* cyclizes, rather than cleaves, lipids (69).

A number of regulatory genes are in Class 1, including putative protein kinases *NIT5* and *HOG2*, protein phosphatase *GAC1*, and the G protein coupled receptor *gprM*. In addition, there are nine TFs: *ZNC1*, *YAP1*, *sclB*, *nosA*, *STU1*, *PAC2*, *FBC1*, and two TFs of unknown function (ucsf_hc.01_1.G217B.00081 and ucsf_hc.01_1.G217B.09028). In the dimorphic fungus *Yarrowia lipolytica*, Znc1 is a negative regulator of hyphal growth (70), and sclB is a known developmental regulator in *Aspergillus*. Of 408 differential genes from RNAseq of *sclB^-^*/WT (71), 31 sclB-repressed and 68 sclB-induced genes have *Histoplasma* orthologs observed in our data. Class 1 contains none of the repressed and five of the induced genes; namely the TFs *FBC1* and ucsf_hc.01_1.G217B.00081 and three enzymes: ucsf_hc.01_1.G217B.03634, ucsf_hc.01_1.G217B.01297, and ucsf_hc.01_1.G217B.08874. Orthologs of *nosA*, *FBC1* and *STU1* are likewise known developmental regulators in *Aspergillus* (72–74), and in *Histoplasma*, *STU1* overexpression is sufficient for hyphal growth at 37°C (11, 12).

To determine if cAMP analog/butyrate addition affects known regulators of yeast-phase growth, we examined our transcriptional profiles for levels of *RYP1-4* transcripts. *RYP1* and *RYP4* transcript levels were not affected by cAMP analogs but were reduced in butyrate-treated samples; *RYP2* transcript levels were reduced in response to cAMP analogs only in one of the two experiments; and *RYP3* levels were not affected by any treatments. Since Ryp1 and Ryp2 are known to be post-transcriptionally regulated (3), we reasoned that cAMP and/or butyrate could affect Ryp protein levels. We performed Western blot analysis to assay the levels of the Ryp1, Ryp2, and Ryp3 proteins in cultures grown in the presence of dbcAMP, 8-CPT-cAMP, or butyrate. Ryp1 and Ryp2 protein levels appeared to be unchanged in response to these chemicals. In contrast, Ryp3 levels were reduced in response to dbcAMP and 8-CPT-cAMP and further reduced in response to butyrate (Fig. 2F). These observations suggest that a reduction in Ryp3 levels may contribute to filamentation in response to these chemical perturbations.

### The WOPR TF *PAC2* and the C2H2 TF *FBC1* are necessary for cAMP-induced filamentation

Our transcriptional analysis indicated that nine TFs change in expression in our data set of interest (Fig. S4). We chose to focus on three of these TFs, all of which have orthologs that affect morphology and basic biology of other fungi. We chose the APSES TF *STU1*, which has previously been shown to affect aerial hyphae formation in *Histoplasma* (13) and whose ectopic expression stimulates inappropriate filamentation at 37°C (11, 12). *STU1* is homologous to the *C. albicans* TF *EFG1*. In *C. albicans*, cAMP production in response to diverse filamentation cues has been shown to activate PKA, in turn activating Efg1 to promote filamentous growth and repress the transcriptional program governed by TFs such as the WOPR TF White-opaque regulator 1 (Wor1) (31). In addition, we subjected the WOPR TF *PAC2*, whose ortholog in *Schizosaccharomyces pombe* has a role in repression of mating (75), to further study. We also examined the role of *FBC1*, a C2H2 TF orthologous to *Aspergillus* spp. FlbC, which is necessary for appropriate conidiation (74).

**Fig 4:**
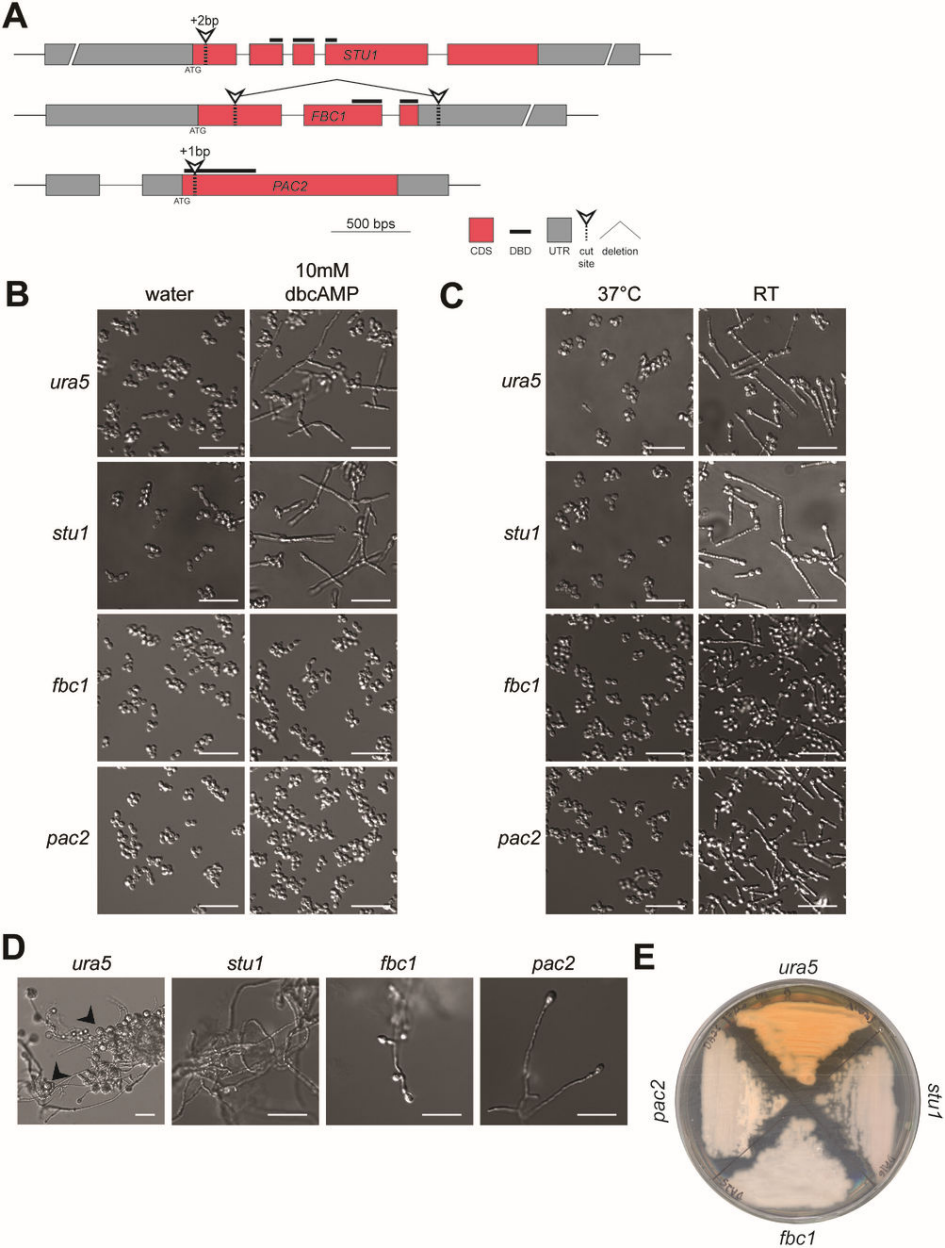
Transcription factor *FBC1* is necessary for RT-induced filamentation. (**A**) Scheme summarizing genetic manipulations of *Histoplasma* using CRISPR/Cas9 to disrupt *STU1* and *PAC2* and delete the open reading frame of *FBC1*. (**B**) Cell morphology of G217B*ura5* (parental strain), *stu1*, *pac2*, and *fbc1 Histoplasma* after 2 days of growth at 37°C in liquid HMM/GlcNAc with or without 10 mM dbcAMP. (**C**) Cell morphology of G217B*ura5*, *stu1*, *pac2*, and *fbc1 Histoplasma* after 8 days of growth at RT in liquid HMM/GlcNAc. (**D**) Cell morphology of G217B*ura5* (parental strain), *stu1*, *pac2*, and *fbc1 Histoplasma* after 8 days of growth at RT on solid Sabouraud media. Arrows indicate macroconidia. (**E**) Gross growth morphology of G217B*ura5* (parental strain), *stu1*, *pac2*, and *fbc1 Histoplasma* after 14 days of growth at RT on solid HMM/GlcNAc media. bp, base pair; CDS, coding sequence; DBD, DNA binding domain; UTR, untranslated region; scale bar denotes 10 µm.

To test whether *STU1*, *FBC1*, or *PAC2* is necessary for filamentation in response to exogenous cAMP or in response to RT, we used CRISPR/Cas9 technology (76–78) to delete the open reading frame of these genes, or to disrupt them by introducing an indel, causing a frame shift and premature termination (Fig. 4A). The *stu1* mutant exhibited filamentous growth in response to dbcAMP or to RT, much like the parental wild-type strain (Fig. 4B and C), indicating that *STU1* is not necessary for dbcAMP- or RT-induced filamentation in liquid media. In contrast, neither the *pac2* nor the *fbc1* mutant produced hyphae when exposed to dbcAMP, indicating that dbcAMP-induced filamentation is dependent on each of these TFs. Interestingly, while the *pac2* mutant was able to transition to hyphal growth within 8 days of RT growth, the *fbc1* mutant strain exhibited a substantial decrease in filamentation in response to RT (Fig. 4C), indicating that *FBC1*, but not *PAC2*, is necessary for RT-induced filamentation. A similar pattern was observed with respect to filamentation in response to butyrate, in which *FBC1* was necessary for full filamentation in response to 10 mM butyrate, while *PAC2* and *STU1* were dispensable (Fig. S5A).

Since fungi exhibit morphologic changes on solid medium, we examined the phenotype of G217B*ura5* (parental strain), *stu1*, *pac2*, and *fbc1* strains on solid Sabouraud media at RT. We observed that the parental strain generated large amounts of macroconidia (Fig. 4D). In contrast, the *stu1*, *pac2*, and *fbc1* mutants were each deficient in the formation of these cells (Fig. 4D). Additionally, when grown on HMM/GlcNAc plates at RT, these mutants yielded glossy, pink colonies as opposed to the fuzzy orange-brown colonies of the parental G217B*ura5* (Fig. 4E), although both colonies consisted of filamentous cells (data not shown). Taken together, these data indicate that each of these TFs is required for the normal developmental program, including macroconidia formation and pigment production, on solid medium at RT.

### Ectopic expression of *PAC2* and *FBC1* stimulates inappropriate filamentation at 37°C

**Fig 5:**
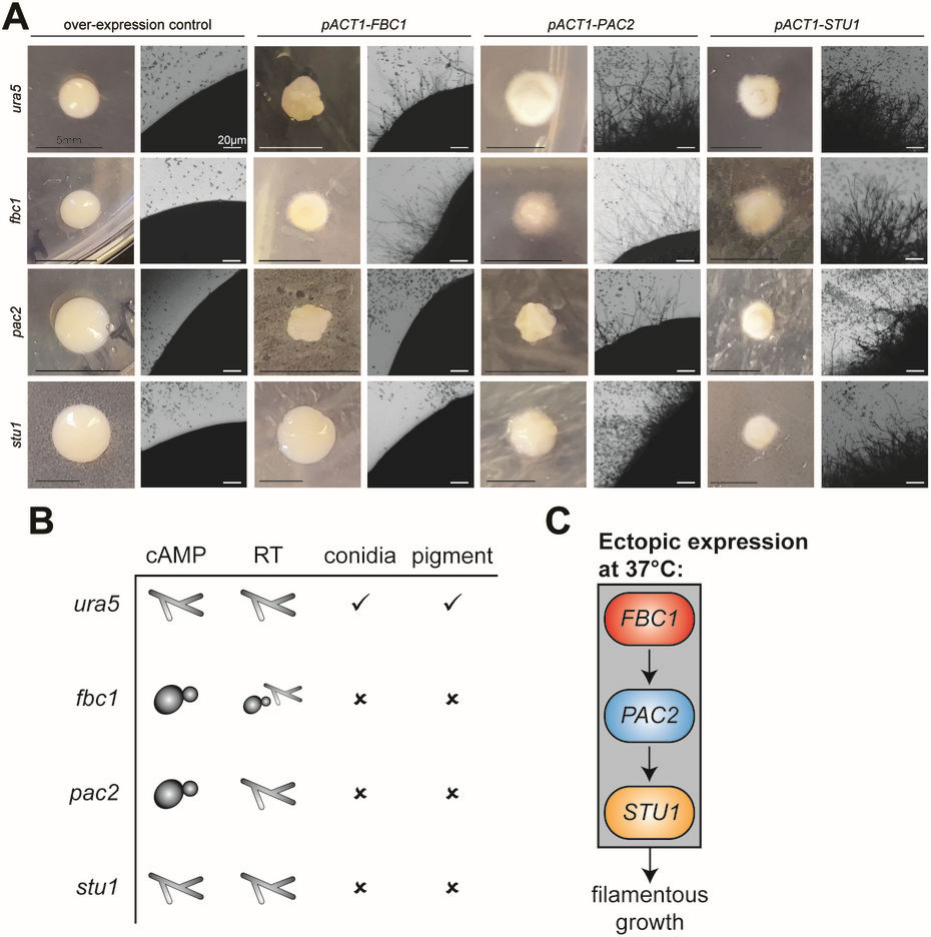
Expression of *PAC2* and *FBC1* is sufficient to trigger inappropriate filamentation at 37°C. (**A**) Various *Histoplasma* strains (genotype indicated on the left) were transformed with ectopic expression constructs carrying *FBC1, PAC2, or STU1* (indicated on the top). For each transformation, a macroscopic image of colonies on the transformation plate (HMM glucose without uracil) is shown on the left, and a microscopic image of the corresponding colony edge is shown on the right. Untransformed microcolonies can be seen as small dark dots in the background of the microscopic images. (**B**) Table summarizing the *fbc1*, *pac2*, and *stu1* phenotypes. (**C**) A diagram showing the genetic relationship between *FBC1*, *PAC2*, and *STU1* in the context of ectopic expression at 37°C.

Ectopic expression of *STU1* is sufficient to override the yeast program and promote robust filamentation at 37°C (11, 12). To test whether deregulated expression of *PAC2* and *FBC1* has a similar phenotype at 37°C, we used a constitutive *ACT1* promoter to drive their expression. Ectopic expression of *PAC2* resulted in >95% filamentous colonies at 37°C, while ectopic expression of *FBC1* resulted in 70% of colonies having an aberrant wrinkled appearance at 37°C corresponding to the presence of both yeast and hyphae (Fig. 5A). To understand the relationships between the activity of the three TFs, we also ectopically expressed each one of them in the background of the three mutants. Overexpression of *FBC1* in the *stu1* mutant did not result in filamentation at 37°C, and overexpression of *PAC2* in the *stu1* mutant resulted in all colonies having a mixed yeast-hyphae morphology. In contrast, ectopic expression of *STU1* in either the *fbc1* or *pac2* mutants triggered filamentation in all transformants at 37°C, suggesting that Stu1 functions downstream of Fbc1 and Pac2. In contrast, ectopic expression of *FBC1* had a much less dramatic phenotype in the *pac2* mutant compared to the parental strain, with all transformants appearing wrinkly but lacking hyphae at the colony edge. These data indicate that the effect of *FBC1* overexpression is dependent on *PAC2*. Conversely, ectopic expression of *PAC2* was able to promote filamentous growth at 37°C in both the *fbc1* (92% of transformants) and *pac2* mutants (81% of transformants) (Fig. 5A). Taken together, these results show that the ability of Fbc1 to induce filamentous growth at 37°C depends on the activity of Pac2 and Stu1, and similarly, Fbc1- and Pac2-dependent filamentation at 37°C depends on Stu1.

## DISCUSSION

In this study, we have shown that exogenous cAMP is sufficient to promote filamentous growth in *Histoplasma* by triggering transcriptional reprogramming at 37°C. We compared the transcriptional response to three filamentation signals—the classic physiological signal of RT, exogenous cAMP at 37°C, and exogenous butyrate at 37°C. We determined that nine TFs are induced by all three of these signals and hypothesized that these factors regulate the hyphal growth program. We focused on three such factors that were induced by cAMP and repressed in the non-filamenting mutant SG1: *STU1*, *FBC1*, and *PAC2*. We have shown that while *STU1* is dispensable for cAMP-induced filamentation, *PAC2* and *FBC1* each are necessary for cAMP-induced filamentation, and *FBC1* is also necessary for robust RT-induced filamentation. Additionally, each of these TFs is necessary for normal hyphal development (including macroconidia and pigment production) on solid media (Fig. 5B). Finally, we have shown that ectopic expression of *STU1*, *FBC1*, or *PAC2* is sufficient to drive filamentous growth at 37°C, that the ability of *FBC1* to promote filamentous growth at 37°C is dependent on *PAC2*, and that the ability of *FBC1* and *PAC2* to promote filamentous growth depends on *STU1*, suggesting the TF hierarchy depicted in Fig. 5C.

We utilized two chemical stimuli that promote filamentation despite growth at 37°C: cyclic AMP and butyrate. While the ability of exogenous cAMP to promote filamentation in *Histoplasma* has been described in the literature (49), the transcriptional changes that occur during cAMP treatment were unknown. Additionally, this study is the first to observe the ability of butyrate to promote filamentous growth. We initially investigated butyrate to determine if its release from dibutyryl-cAMP, a cAMP analog commonly used to study PKA activation, was responsible for the response of *Histoplasma* to dbcAMP. However, the response of *Histoplasma* to butyrate is distinct from its response to dbcAMP: Butyrate is a much more potent filamentation stimulus; it triggers a unique transcriptional response, and the transcriptional response of *Histoplasma* to dbcAMP is near-identical to its transcriptional response to 8-CPT-cAMP, which does not contain butyrate. Thus, we conclude that the effects of dbcAMP are not due to release of butyrate from this compound but rather via its activity in the cAMP pathway. It was informative to utilize butyrate as an orthogonal filamentation stimulus in these studies.

To identify gene sets that correspond with the morphological transition to filamentous growth, we conducted expression profiling of cultures filamenting in response to dbcAMP or to butyrate. About 46% of the total transcriptome is differentially expressed in samples treated with butyrate. Specifically, we detected upregulation of lipid metabolic genes, ergosterol synthesis genes, and ribosomal proteins in response to butyrate. While the upregulation of ribosomal proteins may be connected with the change in the proteome that is necessary to establish a different growth form (yeast vs filaments), the role of lipid metabolism genes in this context remains to be elucidated.

It is possible that the transcriptional response to butyrate reflects a physiologically relevant response to butyrate as a filamentation-inducing signal; for example, in the environment, *Histoplasma* may naturally encounter soil-residing bacteria, which are capable of producing butyrate (79). Interestingly, butyrate can act as a pan-histone deacetylase inhibitor (HDACi) (80). In other fungi, HDACi (including butyrate) have been shown to affect developmental programs: a study conducted in *C. albicans*, *C. parapsilosis*, and *C. neoformans* found that butyrate decreased fungal growth and biofilm formation in *C. albicans* and *C. parapsilosis*, inhibited filamentation in *C. albicans*, and prevented capsule formation and melanization in *C. neoformans* (81). Introduction of butyrate or other HDACi, as well as genetic disruption of HDACs, has also been shown to modulate diverse processes such as virulence, morphology, conidiation, germination, and the expression of SM gene clusters in *Aspergillus*, *Magnaporthe*, *Cryptococcus*, *Cochiobolus carbonum*, and other fungi (82–87).

In contrast to butyrate, the cAMP analogs dbcAMP and 8-CPT-cAMP only affected the expression of 25% of the transcriptome. We did not detect any concerted regulation of carbon utilization pathways in our data in response to cAMP. However, 15 genes involved in amino acid synthesis pathways are upregulated in response to dbcAMP, as well as both subunits of the FAS complex, suggesting that, similar to *S. cerevisiae* (17, 88, 89), *Histoplasma* upregulates biosynthetic pathways in response to cAMP. Interestingly, the Ole1 Δ9-desaturase, which Storlazzi et al. have previously reported to be downregulated in response to exogenous cAMP (90), is upregulated in response to cAMP in our experiments. *OLE1* is not differentially expressed in the steady-state yeast or mycelial phases of *Histoplasma* G217B (3), but appears to be consistently upregulated in the days following the transition of *Histoplasma* cultures to RT (11, 12). These data are contrary to the finding reported by Gargano et al. (91) that *OLE1* transcript was present in G217B yeast but absent in mycelia. Future experiments to target *OLE1* levels could address the function of this gene in morphogenesis. For example, an increase in Ole1 activity could lead to elevated levels of unsaturated fatty acids in cellular membranes, with potential consequences on signaling cascades, thermotolerance, and virulence.

By comparing differential expression induced by both cAMP analogs and butyrate with existing data for differential expression between 37°C and RT and between WT *Histoplasma* and the non-filamenting SG1 mutant, we arrived at high-confidence filamentation- and yeast-associated gene groups. By definition, these groups are well-correlated with previously defined morphology-associated groups that compared gene expression in response to RT with that of SG1 (11, 12). The stringent yeast group is enriched for Ryp1, Ryp2, and Ryp3 direct targets and contains several genes encoding small, secreted proteins and genes involved in iron acquisition, all hallmarks of the *Histoplasma* yeast phase. Interestingly, this group is largely devoid of genes encoding known regulators and TFs. An exception is Mea1, the ortholog of the *A. nidulans* nitrogen regulator MeaB, which is involved in the regulation of nitrogen metabolism (92).

In contrast, the stringent gene group associated with filamentous growth contains multiple transcripts encoding signaling molecules and TFs, as well as transcripts that suggest an upregulation of the pentose phosphate pathway (*ZWF1* and *SHB17*), lipid biosynthesis (*FAS1*, *FAS2*, and *GIG30*), and lipid processing (phospholipases B and D). A connection between cell morphology and the metabolic state of *Histoplasma* remains to be elucidated in future studies.

The stringent hyphal-associated gene group contains nine genes predicted to function as TFs, of which six have previously been associated with regulation of morphology and development in other fungi: the Znc1 ortholog in *Y. lipolytica* regulates filamentation (70); sclB regulates conidiation and SM production in *A. nidulans* (71); *STU1* encodes an APSES TF which was shown by Longo et al. to be necessary for mycelia development of aerial hyphae and conidia in *Histoplasma* (13), and its orthologs stuA and Efg1 regulate development and morphology in *Aspergillus* and *Candida*, respectively (16, 36, 73); *FBC1* ortholog *flbC* regulates conidiation in *Aspergillus* (74); *NosA* regulates sexual development in *A. nidulans* (72); and *PAC2* encodes a WOPR family TF, orthologous to *S. pombe pac-2*, which represses the cAMP-dependent expression of *ste-11*, thus inhibiting mating (75). Taken together, this group of TFs are attractive candidates for regulators that are relevant to the ability of *Histoplasma* to switch between the yeast and hyphal morphology.

We chose to further characterize the role of three of these TFs in promoting filamentation in response to cAMP and RT: *FBC1*, PAC2, and *STU1*. We found that *FBC1* was necessary for robust filamentation in response to cAMP, butyrate, or RT, whereas PAC2 was only necessary for cAMP-induced filamentation and dispensable for butyrate or RT-induced filamentation. *STU1* was not necessary for filamentation in response to cAMP, butyrate, or RT. Based on these data, we hypothesize that filamentation in liquid media in response to RT involves multiple redundant pathways, one of which is induced by an increase in intracellular cAMP and requires *PAC2*. The morphological response of the *fbc1*, *pac2*, and *stu1* mutants to butyrate follows the response pattern to RT; likewise, the changes in the transcriptional profiles of *Histoplasma* in response to RT and in response to butyrate are moderately correlated (*r* ≈ 0.5; Fig. S5B). Finally, it is notable that although *pac2* and *stu1* are able to filament at RT, they show developmental defects in the full hyphal program at RT, as does *fbc1*, for example, conidia formation and pigment production (Fig. 4D and E; summarized in Fig. 5B), indicating that they regulate other RT-induced developmental programs. Additional work is needed to determine whether *FBC1*, *PAC2*, *STU1*, and other TFs whose expression is correlated with filamentous growth act redundantly to drive filamentation in liquid culture in response to temperature.

Our ectopic expression work uncovered interesting relationships between Pac2, Fbc1, and Stu1. We found that *PAC2* was necessary for *FBC1*-driven filamentation, and similarly, *STU1* was necessary for *FBC1* and *PAC2* ectopic expression to cause filamentous growth at 37°C. Taken together, this suggests a genetic pathway with *PAC2* downstream of *FBC1*, and *STU1* downstream of *PAC2* (Fig. 5C). Regulation of eukaryotic transcription is often complex, and further research is necessary to determine the roles of these three TFs in controlling and coordinating the transcriptional response to drive filamentation. We note that *PAC2*, which we have implicated in the hyphal program in this study, and *RYP1*, which is required for the yeast program, are paralogs, suggesting specialization of these individual TFs in opposing developmental programs. Further exploring the circuits that govern the transition between yeast and hyphae in thermally dimorphic fungi will elucidate the critical basic biology of these pathogens and provide an opportunity to develop molecular strategies for targeted therapeutics.

## MATERIALS AND METHODS

### *H. capsulatum* strains and growth conditions

All strain manipulations were done in *G217Bura5*-DA, which is an isolate of the *Histoplasma* G217B*ura5* (WU15) strain. Strains used in this paper can be found in Table S1. Strain frozen stocks (8% DMSO or 15% Glycerol) were streaked onto *Histoplasma* Macrophage Medium (HMM) agarose plates supplemented with 0.2 mg/mL uracil where necessary. Liquid *Histoplasma* cultures were inoculated from solid media plates into HMM + 100 U/mL Penicillin/Streptomycin (with 0.2 mg/mL of uracil if needed) and were passaged 1:25 every 2–3 days into fresh medium, unless indicated otherwise. The base of HMM is Ham’s F-12 medium, which contains 10 mM glucose. HMM media is normally supplemented with an additional 100 mM glucose. HMM/GlcNAc media contained 100 mM N-acetyl glucosamine (A3286, Sigma Aldrich) in place of the additional 100 mM glucose (referred to here as HMM/GlcNAc). Where mentioned, dibutyryl cyclic adenosine monophosphate sodium (dbcAMP, D0627, Sigma Aldrich), 8-(4-chlorophenylthio)-cAMP sodium (8-CPT-cAMP, ab120424, Abcam), 3-isobutyl-1-methylxanthine (IBMX, ab120840, Abcam), sodium butyrate (B5887, Sigma Aldrich), or cyclic adenosine monophosphate sodium (cAMP, A6885, Sigma Aldrich) were added to cultures after dissolving in autoclaved water and filter sterilizing by passing through a 0.22 MCE filter (Santa Cruz Biotechnology). For 37°C growth, plates were grown in a humidified incubator with 5% CO_2_, and liquid cultures were grown on an orbital shaker at 120–150 rpm. For RT growth, plates were wrapped in parafilm and plastic bags and placed in a 25°C incubator in a biosafety level 3 facility. Liquid RT cultures were grown at ambient temperature (26–28°C) on an orbital shaker at 120 rpm.

### Strains and cloning

Primers used in this study are in Table S2. The SG1 mutant was generated using *Agrobacterium*-mediated insertional mutagenesis as described previously (11, 12). Subcloning was performed in *E. coli* DH5α- or DH10b-derived strains. The *STU1* overexpression strain and the control strain were constructed as described previously (11, 12). *FBC1* and *PAC2* overexpression strains were constructed by amplifying the open reading frame (ORF) and 3′ untranslated region (3′UTR) by PCR, and cloned into pTM1 (a pDONR/Zeo based plasmid) downstream of the ACT1 promoter using Gibson cloning kit (NEB), and then introduced into pDG33 using Gateway cloning (Thermo Fisher Scientific, Waltham, MA, USA). To introduce gene disruption, we used the strategy described previously (76–78). Briefly, a gene-appropriate protospacer was inserted into a self-cleaving element using PCR and cloned into the episomal plasmid pBJ219 (which contains the *HcURA5* gene) using Gateway cloning. The plasmid was linearized and introduced into *Histoplasma* G217B*ura5* using electroporation (as described previously in reference 60) along with an mCherry-targeting protospacer as a negative control. Transformants that grew on HMM without uracil were assayed for the disruption using PCR amplifying the targeted genomic region followed by sequencing of the product and indel analysis using the ICE tool (inference of CRISPR edits) from Synthego (93). The colony with the highest indel levels was selected, grown on fresh media, and plated for single colonies. The process was repeated until no wild-type sequence could be detected. The plasmid was then lost by growing the isolate for three passages in liquid media containing uracil, plating the culture for single colonies, and identifying colonies that were auxotrophic for uracil. To create whole gene deletion mutants, the aforementioned technique was adapted to include two protospacers targeting the sequences 5′ and 3′ of the gene. To test for a deletion, DNA was extracted from a liquid *Histoplasma* culture, and PCR with primers external to the deleted sequence, or with primers internal to the sequence, was performed. The colony with the highest amount of editing (short external PCR product, lowest levels of internal PCR product) was selected, grown on fresh media, and plated for single colonies. The process was repeated until no wild-type sequence could be detected.

### Imaging and image analysis

DIC imaging was performed on a Zeiss AxioCam MRM microscope at 40× magnification. Colony images were captured using a Leica microscope. Gross morphology of colonies was captured using a OnePlus 8 Pro camera. To quantify filamentation events, Fiji was used to count yeast and filamentous events in four frames per sample.

### Culture conditions for cAMP expression profiling experiments

Samples were prepared in two separate experiments. In the first experiment, *Histoplasma* G217B*ura5* was grown in liquid HMM culture at 37°C for two passages. On the day of chemical addition, the culture was passaged into fresh HMM/GlcNAc at a final cell density of 6 × 10^6^ cells/mL, and 10 mM dbcAMP, 4 mM 8-CPT-cAMP, 5 mM of sodium butyrate, or filter-sterilized autoclaved water (vehicle control) were added to the appropriate cultures, with three biological replicates for each chemical. Cultures were returned to 37°C growth with shaking. Two days after the addition of each chemical, a small volume of each culture was fixed by adding paraformaldehyde (final paraformaldehyde concentration 4% [vol/vol]), and 10 mL of each culture were harvested by filtration through a 0.45 µm filter using Nalgene Rapid-Flow Sterile Disposable Bottle Top Filters with SFCA Membrane (Fisher 09-740-22G). The cells were scraped from the filter into tubes and promptly flash-frozen in liquid nitrogen.

In the second experiment, *Histoplasma* G217B*ura5* or the SG1 mutant was grown in liquid HMM culture at 37°C for two passages. On the day of dbcAMP addition, the cultures were passaged into fresh HMM/GlcNAc at a final cell density of 6 × 10^6^ cells/mL, and 10 mM dbcAMP or filter-sterilized autoclaved water (vehicle control) was added to the appropriate cultures, with three biological replicates for each chemical-strain combination. The cultures were treated and samples were collected as described for the first experiment.

### RNA extraction

Total RNA was extracted from fungal cells using a Qiazol-based RNA extraction protocol. Frozen cell pellets were resuspended in Qiazol (Qiagen, Germany) and incubated at RT for 5 min to thaw. The lysate was subjected to bead beating (Mini-Beadbeater, Biospec Products, Bartlesville, OK, USA) followed by a chloroform extraction. The aqueous phase was then transferred to an Epoch RNA column where the filter was washed with 3 M NaOAc (pH = 5.5) and then with 10 mM TrisCl (pH = 7.5) in 80% EtOH. DNase (Purelink, Invitrogen, Carlsbad, CA, USA) treatment was used to remove any residual DNA, and the filters were washed again with NaOAc and TrisCl before eluting the RNA in nuclease-free water.

### mRNA isolation

For each experiment, mRNA was extracted from an equal amount of total RNA (up to 20 µg) of each sample. Total RNA samples were treated with TURBO DNase (Thermo Fisher). RNA quality was determined with a RNA 6000 Nano Bioanalyzer chip (Agilent Technologies, Santa Clara, CA, USA). mRNA was purified using polyA selection with Oligo-dT Dynabeads (Thermo Fisher) as described in the manufacturer’s protocol. Ribosomal RNA depletion was confirmed with an RNA 6000 Nano Bioanalyzer chip.

### RNAseq library preparation

Libraries for RNAseq were prepared using the NEB Next Ultra II Directional RNA Library Prep Kit (New England Biolabs, Ipswich, MA, USA). Individual libraries were uniquely barcoded with NEBNext Multiplex Oligos for Illumina sequencing platform (New England Biolabs). Average fragment size and presence of excess adapter was determined with High Sensitivity DNA Bioanalyzer chip from Agilent Technologies. Libraries had an average fragment length of 300–500 bp. The concentration of the individual libraries was quantified using the High Sensitivity DNA Qubit assay (Thermo Fisher). A total of 5 ng of each library was pooled into each final library and run on a High Sensitivity DNA Bioanalyzer chip to determine the average fragment size of the final pooled samples. The final libraries were submitted to the UCSF Center for Advanced Technology for sequencing on an Illumina HiSeq 4000 sequencer.

### Transcriptome analysis

Transcript abundances were quantified based on version ucsf_hc.01_1.G217B of the *Histoplasma* G217B transcriptome (S5 Data of reference 3). Relative abundances (reported as TPM values [94]) and estimated counts (est_counts) of each transcript in each sample were estimated by alignment-free comparison of k-mers between the reads and mRNA sequences using KALLISTO version 0.46.0 (95). Further analysis was restricted to transcripts with TPM ≥10 in at least one sample.

Differentially expressed genes were identified by comparing replicate means for contrasts of interest using LIMMA version 3.30.8 (96, 97). Genes were considered significantly differentially expressed if they were statistically significant (at 5% FDR) with an effect size of at least 1.5× (absolute log2 fold change ≥ 0.585) for a given contrast.

For comparison with previous expression profiling, the above KALLISTO/LIMMA pipeline was applied to the reads from reference 3 (GEO accession GSE68706) and LIMMA fit parameters were taken from S2 Data of reference 11. Enrichments for Ryp1–3 targets in gene sets were calculated using a hypergeometric test, followed by Benjamini-Hochberg correction for multiple hypotheses testing, with adjusted *P* < 0.05 considered significant.

### Protein extraction

Organic fractions from Qiazol-chloroform extraction were stored at –20°C. After thawing the fractions, 100% ethanol was added, and samples were centrifuged to pellet DNA. The protein-containing supernatant was added to isopropanol and centrifuged at 4°C to pellet the protein precipitate. The pellet was washed three times with 0.3 M guanidinium thiocyanate in 95% ethanol, followed by centrifugation at 4°C, after which 100% ethanol was added to the pellets. The protein pellets were vortexed and incubated at RT for 20 min, centrifuged at 4°C, and air-dried at RT. The pellets were resuspended in urea lysis buffer (9 M urea, 25 mM Tris-HCl, 1% SDS, and 0.7 M β-mercaptoethanol) and incubated at 50°C up to 20 min until fully dissolved in the buffer, followed by boiling and centrifugation. The supernatant was transferred into a clean tube and quantified using the Pierce 660 nm assay with added ionic detergent compatibility reagent (Thermo Fisher).

### Western blotting

Following quantification of protein, an equal amount per sample of 12 µg was boiled with Novex NuPAGE LDS Sample Buffer (Invitrogen) and loaded onto a 10-well Novex NuPAGE 4–12% BT SDS-PAGE gel (Invitrogen). Electrophoresis was performed in MOPS running buffer at 150 V. The protein was then transferred to a nitrocellulose membrane at approximately 35 V for 2 h. The membrane was incubated with Intercept (PBS) Blocking Buffer (LI-COR, Lincoln, NE) for an hour and then incubated in the primary antibody in wash buffer overnight at 4°C. Polyclonal peptide antibodies against either Ryp1, Ryp2, or Ryp3 were used as primary antibodies in the following dilutions in blocking buffer: rabbit anti-Ryp1 (1:10,000), rabbit anti-Ryp2 (1:2,500), and rabbit anti-Ryp3 (1:5,000) (4). As a loading control, monoclonal mouse anti-α-tubulin (1:1,000) was used (DM1A, Novus Biologicals, Littleton, CO, USA). The blot was washed with PBS + 0.1% Tween-20 three times, and secondary antibody was added to the blot for 1 h at RT: IRDye 800CW goat anti-rabbit IgG, or IRDye 680RD donkey anti-mouse IgG (both 1:10,000, LI-COR). The blot was washed with PBS + 0.1% Tween-20 three times and imaged using the LI-COR Odyssey CLx system.

### *Histoplasma* genomic DNA extraction

The cells from 10 mL of dense liquid culture were collected by centrifugation and kept at –80°C until DNA extraction. The cell pellets were thawed and washed in TE buffer and then resuspended in lysis buffer (50 mM Tris [pH 7.2], 50 mM EDTA, 0.1 M SDS, and 0.14 M β-mercaptoethanol). The cells were lysed in buffer by bead-beating with zirconia beads, and the samples were incubated at 65°C for 1 h. 800 µL of 1:1 phenol-chloroform solution was added to each sample followed by thorough mixing. The samples were centrifuged for 15 min at 13,000 rpm. The aqueous fraction was added to 470 µL of 0.13 M sodium acetate in 95% isopropanol and mixed by inversion to precipitate the DNA. The supernatant was discarded after a 2-min centrifugation, and the pellet was washed with 70% ethanol and air-dried at 65°C. 1 mL TE buffer with 1.5 µL RNase A (10 mg/mL) was added to each sample, and the pellet was dissolved at 65°C.

## Data Availability

All relevant data are contained within the paper and/or supporting information files. For high-throughput sequencing data, the raw data are available at the NCBI Sequence Read Archive (SRA) and Gene Expression Omnibus (GEO) databases under GEO accession GSE218032 (RNAseq) and SRA accession PRJNA1283523 (DNAseq).

## SUPPLEMENTAL MATERIAL

Fig. S1 mbio.02234-25-s0001.tif dbcAMP promotes filamentous growth at 37°C.

Fig. S2 mbio.02234-25-s0002.tif Cellular morphologies of RNAseq samples.

Fig. S3 mbio.02234-25-s0003.tif Classification of genes by expression profile.

Fig. S4 mbio.02234-25-s0004.tif Transcript abundances of transcription factors in the stringently filamentous gene group.

Fig. S5 mbio.02234-25-s0005.tif Transcription factor FBC1 is necessary for butyrate-induced filamentation.

Supplemental material mbio.02234-25-s0006.docx Supplemental figure and table legends; Tables S1 and S2.

Table S3 mbio.02234-25-s0007.txt Heatmap showing 5,494 genes differentially expressed in response to butyrate, dbcAMP, or 8-CPT-cAMP.

Table S4 mbio.02234-25-s0008.txt Gene expression ratios.

Table S5 mbio.02234-25-s0009.txt Yeast regulon.

Table S6 mbio.02234-25-s0010.txt Hyphal regulon.

ASM does not own the copyrights to Supplemental Material that may be linked to, or accessed through, an article. The authors have granted ASM a non-exclusive, world-wide license to publish the Supplemental Material files. Please contact the corresponding author directly for reuse.
