# Supplementary figures and images for: Chemical stimuli override a temperature-dependent morphological program by reprogramming the transcriptome of a fungal pathogen
Source: mBio. 2025 Sep 10;16(10):e02234-25. doi: 10.1128/mbio.02234-25 (PMC12505909; doi:10.1128/mbio.02234-25)

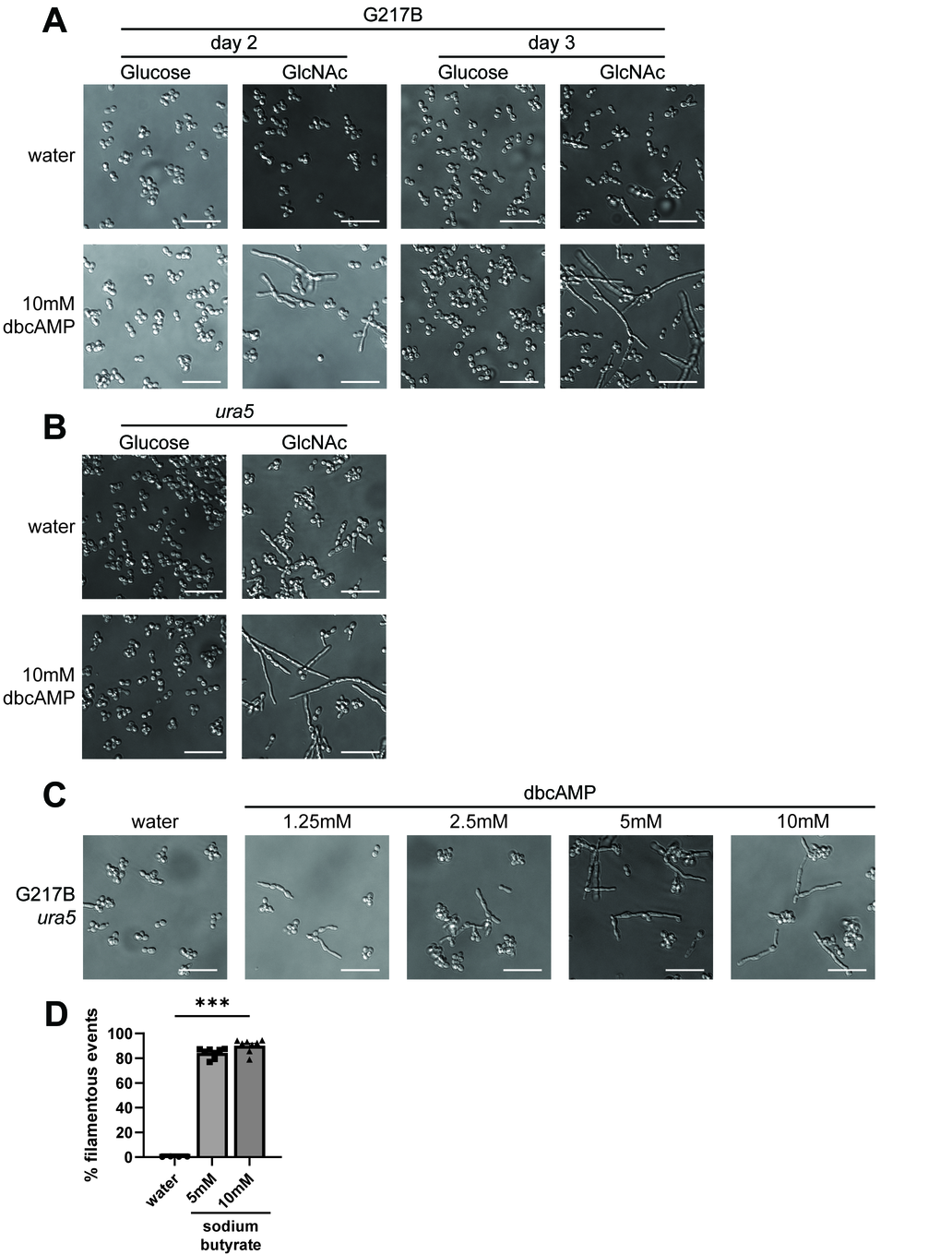

Supplement: Fig. S1 — dbcAMP promotes filamentous growth at 37°C. [file mbio.02234-25-s0001.tif]

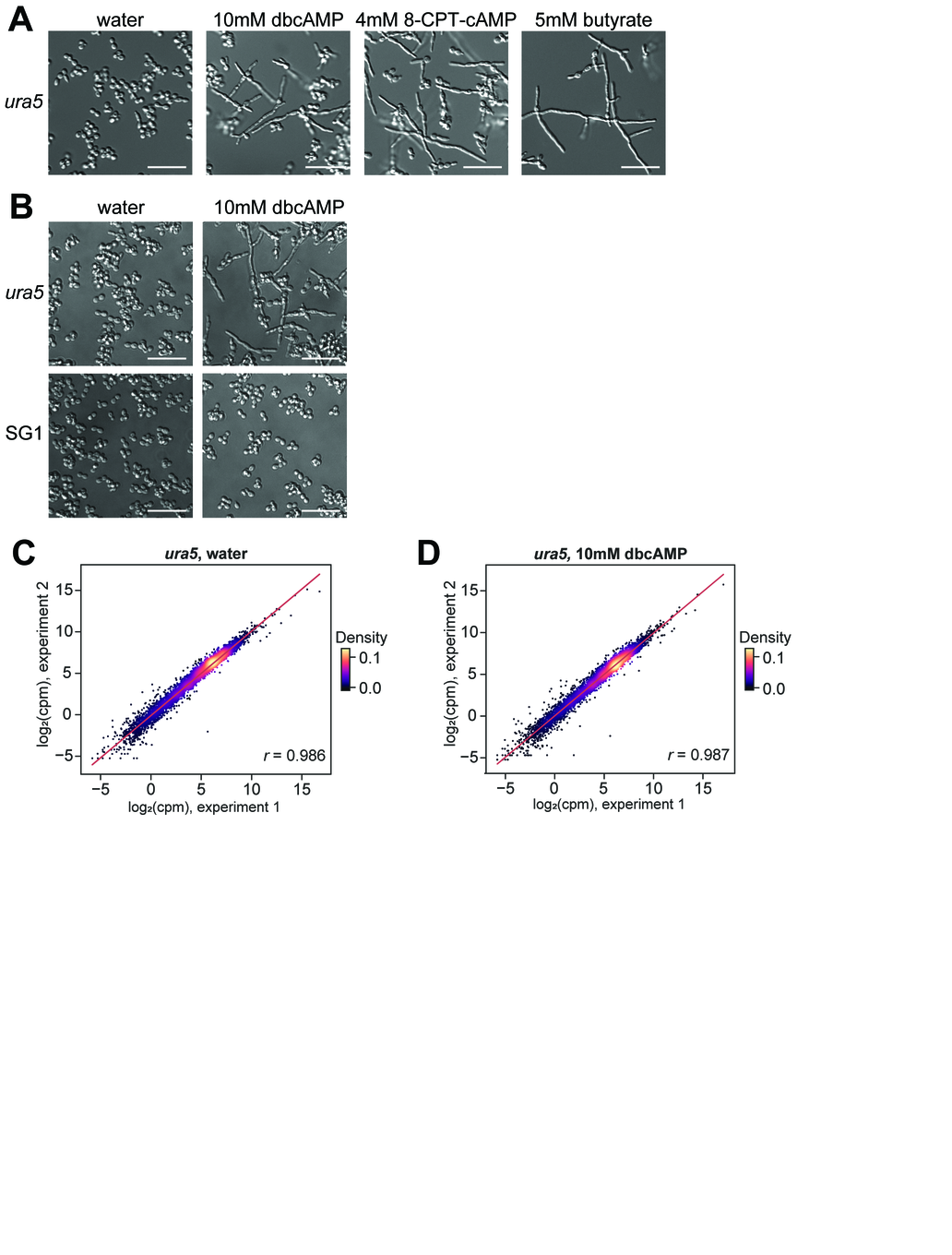

Supplement: Fig. S2 — Cellular morphologies of RNAseq samples. [file mbio.02234-25-s0002.tif]

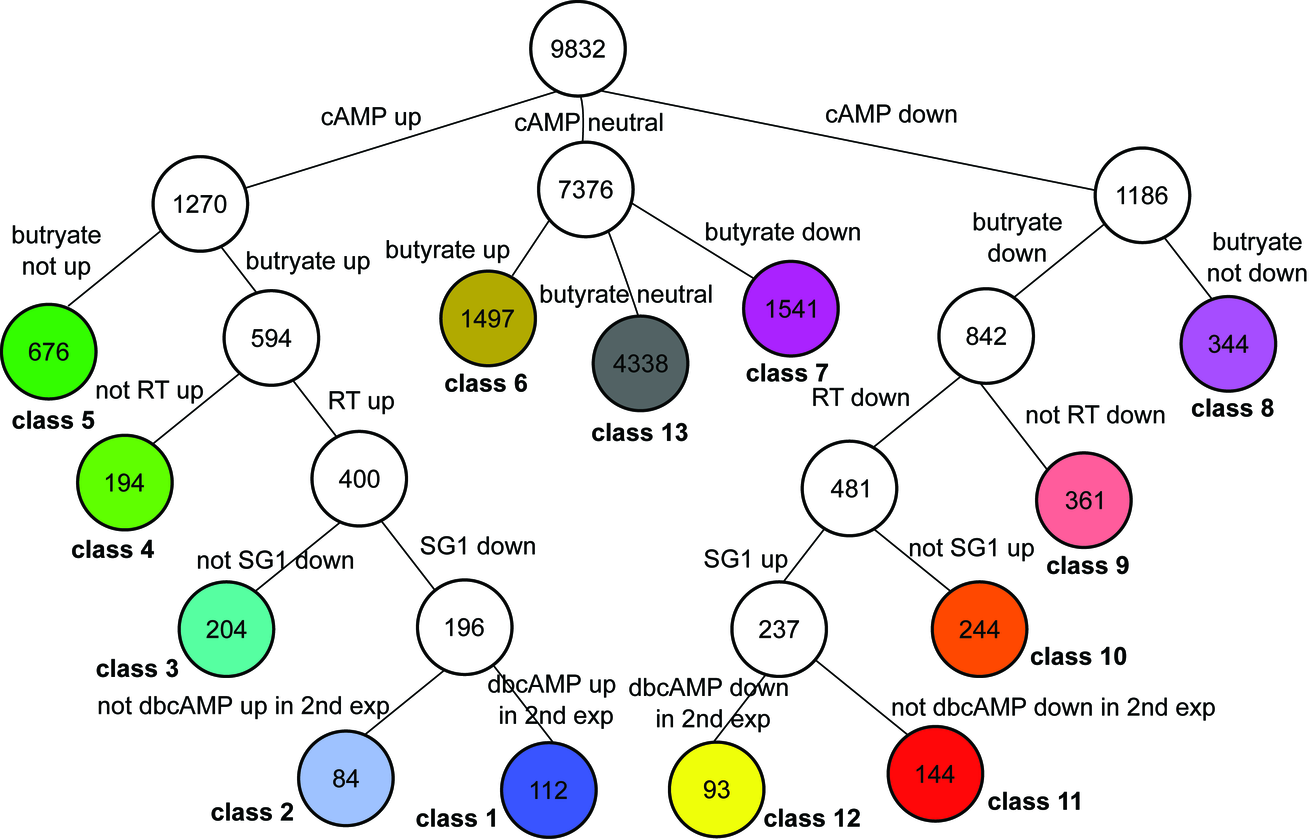

Supplement: Fig. S3 — Classification of genes by expression profile. [file mbio.02234-25-s0003.tif]

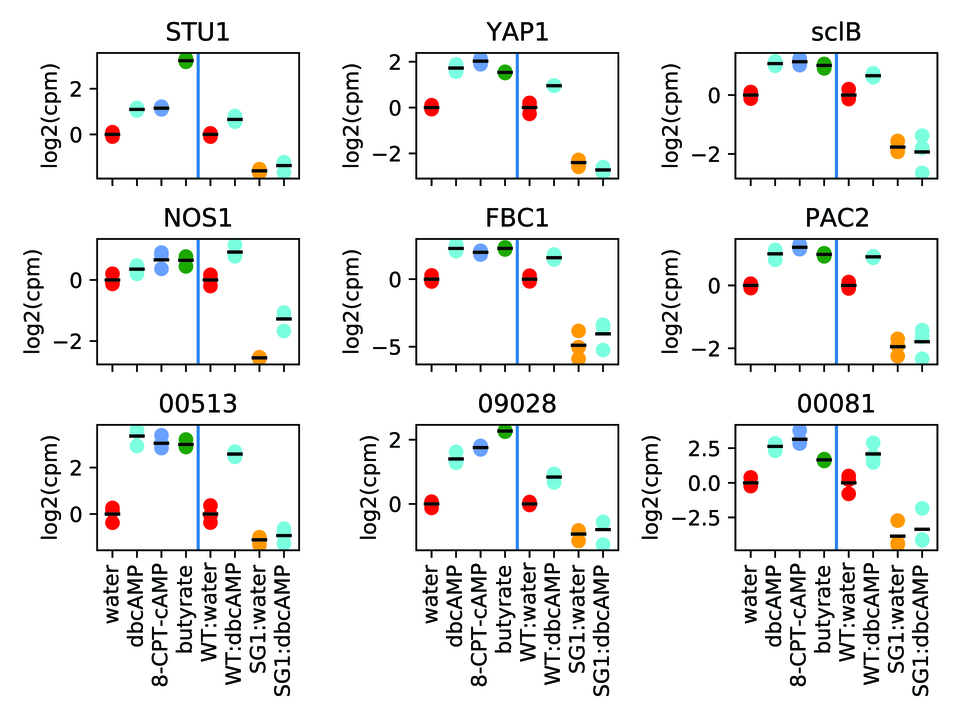

Supplement: Fig. S4 — Transcript abundances of transcription factors in the stringently filamentous gene group. [file mbio.02234-25-s0004.tif]

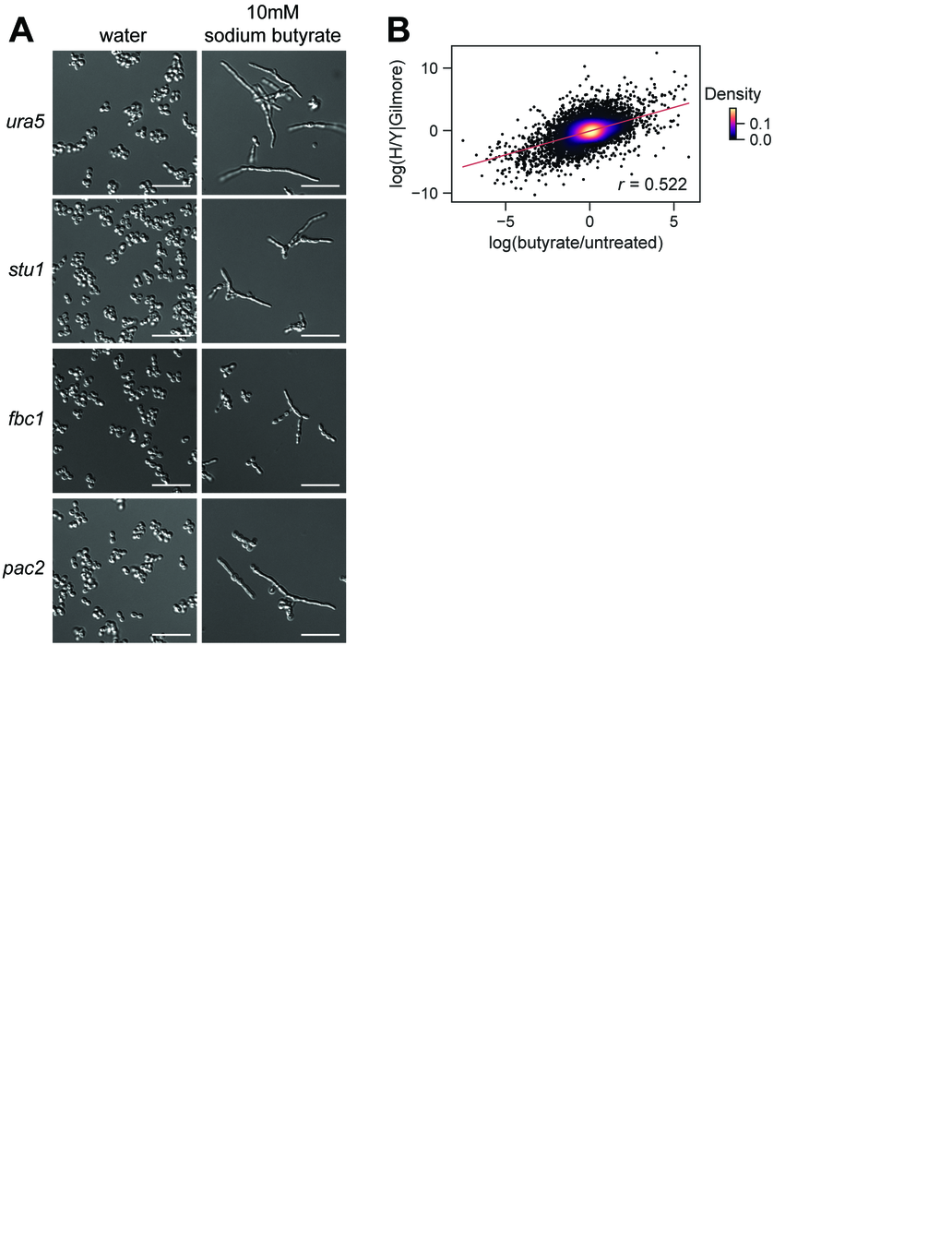

Supplement: Fig. S5 — Transcription factor FBC1 is necessary for butyrate-induced filamentation. [file mbio.02234-25-s0005.tif]
