# Supplementary material for: Chemical stimuli override a temperature-dependent morphological program by reprogramming the transcriptome of a fungal pathogen
Source: mBio. 2025 Sep 10;16(10):e02234-25. doi: 10.1128/mbio.02234-25 (PMC12505909; doi:10.1128/mbio.02234-25)
Supplement: Supplemental material — Supplemental figure and table legends; Tables S1 and S2. [file mbio.02234-25-s0006.docx]

**Supplemental Figure Legends**

**Supplemental Figure 1. dbcAMP promotes filamentous growth at 37°C.** (A) Cell morphology of G217B *Histoplasma* after 2 and 3 days of growth at 37°C in liquid HMM or HMM/GlcNAc with or without 10mM dbcAMP. (B) Cell morphology of G217B*ura5* *Histoplasma* after 3 days of growth at 37°C in liquid HMM or HMM/GlcNAc with or without 10mM dbcAMP. (C) Cell morphology of G217B*ura5 Histoplasma* after 2 days of growth at 37°C in liquid HMM/GlcNAc with various concentrations of dbcAMP. (D) Quantification of filamentation after 2 days at 37°C in liquid HMM/GlcNAc with 5mM or 10mM sodium butyrate, or water as vehicle. *** p < 0.001, Kruskal-Wallis test. Scale bar denotes 10μm.

**Supplemental Figure 2. Cellular morphologies of RNAseq samples.** (A) Cell morphology of G217B*ura5 Histoplasma* after 2 days of growth at 37°C in liquid HMM/GlcNAc with 10mM dbcAMP, 4mM 8-CPT-cAMP, 5mM butyrate, or water as vehicle control. (B) Cell morphology of G217B*ura5* and SG1 *Histoplasma* after 2 days of growth at 37°C in liquid HMM/GlcNAc with 10mM dbcAMP or water as vehicle control. (C, D) Scatter plots of log_2_(cpm) values in corresponding conditions between the first and the second experiment. Scale bar denotes 10μm. r, Pearson’s correlation coefficient.

**Supplemental Figure 3. Classification of genes by expression profile.** Scheme for the classification of genes as discussed in the text and shown in Fig. 3. The top node indicates the 9832 genes observable in the expression profiling data. Edges divide each internal node into disjoint sets, with edge labels indicating the division criteria. Numbers inside nodes indicate the number of genes in the corresponding set. The colored terminal nodes indicate the final, disjoint classification spanning the 9832 observed genes.

**Supplemental Figure 4.** **Transcript abundances of transcription factors in the stringently filamentous gene group.** Transcript abundances of transcription factors in the stringently filamentous gene group (class 1 in Figure 3A), reported as log_2_ of read counts per million (cpm) in two separate experiments (as indicated by the two halves of each box). The conditions for the left half of each box are water, dbcAMP, 8-CPT-cAMP and butyrate for the wild-type strain. Conditions for the right half of each box are either water or dbcAMP for either the wild-type or SG1 strain, as indicated. Short black lines represent LIMMA fit values of transcript abundances for each condition. The transcript abundances are normalized to the wild-type, water LIMMA fit values of their respective experiment. Each box is labeled with the name of the corresponding TF or a ucsf_hc.01_1.G217B transcript number for unannotated TFs (S5 Data of (3)).

**Supplemental Figure 5. Transcription factor *FBC1* is necessary for butyrate-induced filamentation.** (A) Cell morphology of G217B*ura5*, *stu1*, *pac2*, and *fbc1* *Histoplasma* after 2 days of growth at 37°C in liquid HMM/GlcNAc with or without 10mM sodium butyrate. (B) Scatter plot of LIMMA-fit butyrate/vehicle contrast vs. hyphae/yeast contrast log2-ratios from Table S4. Scale bar denotes 10μm.

**Supplemental Tables S1 and S2 (S3, 4, 5, 6 uploaded separately)**

# Table S1: Strains used in this study

| Name | Referred in this study as | Comment |
| --- | --- | --- |
| G217B-DA | G217B | Derived from ATCC 26032 through passaging. |
| G217B*ura5*-DA | G217B*ura5* or *ura5* | Derived from WU15 through passaging; parental strain used to generate CRISPR mutants and over-expression strains. |
| G217B*ura5* *msb2*::T-DNA | SG1 | WU15 with T-DNA insertion upstream of *MSB2* ORF (11). |
| G217B*ura5*-DA *fbc1*Δ | *fbc1* | Deletion strain of *FBC1* (ATG+273..1555 deleted). |
| G217B*ura5*-DA *pac2-1* | *pac2* | Disruption strain of *PAC2* (1 bp insertion in position ATG+33). |
| G217B*ura5*-DA *stu1-2* | *stu1* | Disruption strain of *STU1* (2 bp insertion in position ATG+26). |
| G217B*ura5*-DA p*ACT1*-t*CATB*; *URA5* | *ura5* + Overexpression control | Overexpression control. |
| G217B*ura5*-DA p*ACT1*-*FBC1*-t*CATB*; *URA5* | *ura5* + *pACT1-FBC1* | Overexpression of *FBC1*. |
| G217B*ura5*-DA p*ACT1*-*PAC2*-t*CATB*; *URA5* | *ura5* + *pACT1-PAC2* | Overexpression of *PAC2*. |
| G217B*ura5-DA* p*ACT1*-*STU1*-t*CATB*; *URA5* | *ura5* + *pACT1-STU1* | Overexpression of *STU1*. |
| G217B*ura5*-DA *fbc1*Δ p*ACT1*-t*CATB*; *URA5* | *fbc1* + Overexpression control | Overexpression control in the *fbc1* strain. |
| G217B*ura5*-DA *fbc1*Δ p*ACT1*-*FBC1*-t*CATB*; *URA5* | *fbc1 + pACT1-FBC1* | Overexpression of *FBC1* in the *fbc1* strain. |
| G217B*ura5*-DA *fbc1*Δ p*ACT1*-*PAC2*-t*CATB*; *URA5* | *fbc1 + pACT1-PAC2* | Overexpression of *PAC2* in the *fbc1* strain. |
| G217B*ura5*-DA *fbc1*Δ p*ACT1*-*STU1*-t*CATB*; *URA5* | *fbc1 + pACT1-STU1* | Overexpression of *STU1* in the *fbc1* strain. |
| G217B*ura5*-DA *pac2-1* p*ACT1*-t*CATB*; *URA5* | *pac2* + Overexpression control | Overexpression control in the *pac2* strain. |
| G217B*ura5*-DA *pac2-1* p*ACT1*-*FBC1*-t*CATB*; *URA5* | *pac2 + pACT1-PAC2* | Overexpression of *FBC1* in the *pac2* strain. |
| G217B*ura5*-DA *pac2-1* p*ACT1*-*PAC2*-t*CATB*; *URA5* | *pac2 + pACT1-PAC2* | Overexpression of *PAC2* in the *pac2* strain. |
| G217B*ura5*-DA *pac2-1* p*ACT1*-*STU1*-t*CATB*; *URA5* | *pac2 + pACT1-STU1* | Overexpression of *STU1* in the *pac2* strain. |
| G217B*ura5*-DA *stu1-2* p*ACT1*-t*CATB*; *URA5* | *stu1* + Overexpression control | Overexpression control in the *stu1* strain. |
| G217B*ura5*-DA *stu1-2* p*ACT1*-*FBC1*-t*CATB*; *URA5* | *stu1 + pACT1-FBC1* | Overexpression of *FBC1* in the *stu1* strain. |
| G217B*ura5*-DA *stu1-2* p*ACT1*-*PAC2*-t*CATB*; *URA5* | *stu1 + pACT1-PAC2* | Overexpression of *PAC2* in the *stu1* strain. |
| G217B*ura5*-DA *stu1-2* p*ACT1*-*STU1*-t*CATB*; *URA5* | *stu1 + pACT1-STU1* | Overexpression of *STU1* in the *stu1* strain. |

# Table S2: Primers used in this study

| Number | Sequence | Purpose | Template |
| --- | --- | --- | --- |
| ***Primers for cloning of the STU1 CRISPR/Cas9 plasmid*** | | | |
| 5699 | ATGGCAGAGCTCCAGTCATC | Amplify the 5’ segment of gRNA cassette. | pPTS608-Cas9-hyg-Pra1-sgRNA (77) |
| 7351 | GACGAGCTTACTCGTTTCGTCCTCACGGACTCATCAGAATGTGCGGTGATGTCTGCTCAAGC | Amplify the 5’ segment of gRNA cassette. Underlined: gene specific sequence. | pPTS608-Cas9-hyg-Pra1-sgRNA |
| 5702 | TTTGCTTTTCCCGAACTT | Amplify the 3’ segment of gRNA cassette. | pPTS608-Cas9-hyg-Pra1-sgRNA |
| 7350 | GGACGAAACGAGTAAGCTCGTCAATGTGATGCATGTACATCCGTTTTAGAGCTAGAAATAGCAAG | Amplify the 3’ segment of gRNA cassette. Underlined: gene specific sequence. | pPTS608-Cas9-hyg-Pra1-sgRNA |
| 5769 | GGGGACAAGTTTGTACAAAAAAGCAGGCTGCGTAAGCTCCCTAATTGGC | Fusing the 5’ and 3’ segments and adding attB sites. | 5’ + 3’ segments |
| 5770 | GGGGACCACTTTGTACAAGAAAGCTGGGTGAGCCAAGAGCGGATTCCT | Fusing the 5’ and 3’ segments and adding attB sites. | 5’ + 3’ segments |
| ***Primers for amplification and testing the efficiency of the CRISPR/Cas9 disruption of STU1*** | | | |
| 6326 | CCGTGCCCAGTTATACGACG | To sequence the *STU1* disruption site. | G217B *STU1* locus |
| 3109 | GCACAGCAATCCTCCTCTTC | To amplify *STU1* around disruption site. | G217B *STU1* locus |
| 6327 | CATCGGCCCTATTTTGACGACAT | To amplify *STU1* around disruption site. | G217B *STU1* locus |
| ***Primers for cloning of the FBC1 CRISPR/Cas9 plasmid*** | | | |
| 6631 | *GCCCGGGC*TAACTTGTTGCGTTCC | Amplify the 5’ segment of gRNA cassette. Italicized: SrfI site. | pPTS608-Cas9-hyg-Pra1-sgRNA |
| 7369 | GACGAGCTTACTCGTTTCGTCCTCACGGACTCATCAGCAGGGTCGGTGATGTCTGCTCAAGC | Amplify the 5’ segment of gRNA cassette. Underlined: gene specific sequence. | pPTS608-Cas9-hyg-Pra1-sgRNA |
| 7371 | GACGAGCTTACTCGTTTCGTCCTCACGGACTCATCAGACCCCACGGTGATGTCTGCTCAAGC | Amplify the 5’ segment of gRNA cassette. Underlined: gene specific sequence. | pPTS608-Cas9-hyg-Pra1-sgRNA |
| 5708 | GAGCCAAGAGCGGATTCCT | Amplify the 3’ segment of gRNA cassette. | pPTS608-Cas9-hyg-Pra1-sgRNA |
| 7368 | GGACGAAACGAGTAAGCTCGTCCAGGGTTGTAATTACTGCTCGTTTTAGAGCTAGAAATAGCAAG | Amplify the 3’ segment of gRNA cassette. Underlined: gene specific sequence. | pPTS608-Cas9-hyg-Pra1-sgRNA |
| 7370 | GGACGAAACGAGTAAGCTCGTCACCCCAACATGCTTACGGGGGTTTTAGAGCTAGAAATAGCAAG | Amplify the 3’ segment of gRNA cassette. Underlined: gene specific sequence. | pPTS608-Cas9-hyg-Pra1-sgRNA |
| 6630 | GGGGACAAGTTTGTACAAAAAAGCAGGCT*GCCCGGGC*TAACTTGTTG | Fusing the 5’ and 3’ segments of gRNA cassette A and adding attB sites and SrfI site. | 5’ + 3’ segments of cassette A |
| 5770 | GGGGACCACTTTGTACAAGAAAGCTGGGTGAGCCAAGAGCGGATTCCT | Fusing the 5’ and 3’ segments of gRNA cassette A and adding attB sites. | 5’ + 3’ segments of cassette A |
| 6329 | CGCAACAAGTTAGCCCGAGCCAAGAGCGGATTCCT | Fusing the 5’ and 3’ segments of gRNA cassette B and adding a SrfI site. | 5’ + 3’ segments of cassette B |
| 6380 | AAAAGCAGGCTGCCC*GCCCGGGC*TAACTTGTTG | Fusing the 5’ and 3’ segments of gRNA cassette B and adding a SrfI site. | 5’ + 3’ segments of cassette B |
| ***Primers for amplification and testing the efficiency of the CRISPR/Cas9 deletion of FBC1*** | | | |
| 7402 | GCATCCCTCTTGTTTTCTTGTC | To amplify outside of the FBC1 deletion | G217B *FBC1* locus |
| 7411 | GAAGAAAAACTTATGAAGCCGTACCGTC | To amplify outside of the FBC1 deletion | G217B *FBC1* locus |
| 7406 | GACTACCTTTGATCAAGCCCAAG | To amplify a sequence inside of the FBC1 deletion | G217B *FBC1* locus |
| 7408 | TGGAGCTGTAGTCGGTGACG | To amplify a sequence inside of the FBC1 deletion | G217B *FBC1* locus |
| ***Primers for cloning of the PAC2 CRISPR/Cas9 plasmid*** | | | |
| 6631 | *GCCCGGGC*TAACTTGTTGCGTTCC | Amplify the 5’ segment of gRNA cassette. Italicized: SrfI site. | pPTS608-Cas9-hyg-Pra1-sgRNA |
| 7373 | GACGAGCTTACTCGTTTCGTCCTCACGGACTCATCAGCGGACACGGTGATGTCTGCTCAAGC | Amplify the 5’ segment of gRNA cassette. Underlined: gene specific sequence. | pPTS608-Cas9-hyg-Pra1-sgRNA |
| 5708 | GAGCCAAGAGCGGATTCCT | Amplify the 3’ segment of gRNA cassette | pPTS608-Cas9-hyg-Pra1-sgRNA |
| 7372 | GGACGAAACGAGTAAGCTCGTCCGGACACGTCCGCACACCCGGTTTTAGAGCTAGAAATAGCAAG | Amplify the 3’ segment of gRNA cassette. Underlined: gene specific sequence. | pPTS608-Cas9-hyg-Pra1-sgRNA |
| 6630 | GGGGACAAGTTTGTACAAAAAAGCAGGCT*GCCCGGGC*TAACTTGTTG | Fusing the 5’ and 3’ segments of gRNA cassette and adding attB sites and SrfI site. | 5’ + 3’ segments |
| 5770 | GGGGACCACTTTGTACAAGAAAGCTGGGTGAGCCAAGAGCGGATTCCT | Fusing the 5’ and 3’ segments of gRNA cassette and adding attB sites. | 5’ + 3’ segments |
| ***Primers for amplification and testing the efficiency of the CRISPR/Cas9 disruption of PAC2*** | | | |
| 7369 | CCGTTATTGTATGACCAGGAG | To amplify *PAC2* around disruption site. | G217B *PAC2* locus |
| 7399 | TGTACAGATCTTTCGTACGG | To amplify *PAC2* around disruption site. | G217B *PAC2* locus |
| 7397 | tagGCTCCGGTTTTGTTTAGG | To sequence *PAC2* disruption site. | G217B *PAC2* locus |
| ***Cloning overexpression plasmids of FBC1 and PAC2*** | | | |
| 7454 | ACCTCGTTAAGTAGCCCACAATGACTATGGTTATCGAAAACCGAAACCG | To amplify the *FBC1* ORF and 3’ UTR | G217B *FBC1* locus |
| 7455 | TATGGTATGAGGTTTGAGGCCGCGCACAGGTATTAATCAAGAG | To amplify the *FBC1* ORF and 3’ UTR | G217B *FBC1* locus |
| 7457 | CTCGTTAAGTAGCCCACAATGGAGACGTATAACGGACACG | To amplify the *PAC2* ORF and 3’ UTR | G217B *PAC2* locus |
| 7456 | CCTCAAACCTCATACCATATGCCCCGATTTGAGTCCTTTTCC | To amplify the *PAC2* ORF and 3’ UTR | G217B *PAC2* locus |
| 7420 | TATGGTATGAGGTTTGAGGCGCAA | To linearize pTM1 between the *ACT1* promoter and the *CATB* terminator | pTM1 (11) |
| 7421 | TGTGGGCTACTTAACGAGGT | To linearize pTM1 between the *ACT1* promoter and the *CATB* terminator | pTM1 |

**Supplemental Table legends**

Table S3. Table of annotations and limma statistics for differentially expressed genes.

Excel-compatible tab-delimited text conforming to JavaTreeView extended CDT format. Each row is a transcript with the UNIQID column giving the systematic gene name from PMID:26177267. Annotation columns are taken from S4 of PMID:35089059: NAME -- manually curated short gene name; Description; HcG217B_pred -- systematic name from the 9/21/2005 predicted gene set from Washington University; HcG217B_acc -- GenBank accession from GCA_017607445.1; HcG217B_rc -- repeat classification as in fig 4A of PMID:35089059. ChIP-chip associations (RypX_chip columns) are taken from S2 of PMID:23935449 based on the HcG217B_pred column. The p(X) columns give limma BH-adjusted p-values for differential expression in each contrast. Class indicates the differential expression pattern, as referenced in the results and discussion sections. BGCOLOR gives the hex code for the class coloring in fig fig:heatmap and fig fig:decision_tree. GWEIGHT is a place-holder column for JavaTreeView compatibility. The final seven columns give the limma fit contrasts as log2(CPM ratio) values.

Table S4. Table of annotations and limma statistics for all expressed genes.

Excel-compatible tab-delimited text conforming to JavaTreeView extended CDT format. Columns are exactly as for S1 Table, but rows include all 9832 analyzed genes.

Table S5. Table of annotations and limma statistics for annotations for class 1 genes.

Excel-compatible tab-delimited text conforming to JavaTreeView extended CDT format. Columns are exactly as for S1 Table, but limited to the 112 class 1 genes.

Table S6. Table of annotations and limma statistics for class 12 genes.

Excel-compatible tab-delimited text conforming to JavaTreeView extended CDT format. Columns are exactly as for S1 Table, but limited to the 93 class 12 genes.
